# Combinatorial Loss of the Enzymatic Activities of Viral Uracil-DNA Glycosylase and Viral dUTPase Impairs Murine Gammaherpesvirus Pathogenesis and Leads to Increased Recombination-Based Deletion in the Viral Genome

**DOI:** 10.1128/mBio.01831-18

**Published:** 2018-10-30

**Authors:** Qiwen Dong, Kyle R. Smith, Darby G. Oldenburg, Maxwell Shapiro, William R. Schutt, Laraib Malik, Joshua B. Plummer, Yunxiang Mu, Thomas MacCarthy, Douglas W. White, Kevin M. McBride, Laurie T. Krug

**Affiliations:** a Department of Molecular Genetics and Microbiology, Stony Brook University, Stony Brook, New York, USA; b Graduate Program of Molecular and Cellular Biology, Stony Brook University, Stony Brook, New York, USA; c Gundersen Health System, La Crosse, Wisconsin, USA; d Department of Applied Mathematics and Statistics, Stony Brook University, Stony Brook, New York, USA; e Department of Computer Science, Stony Brook University, Stony Brook, New York, USA; f Department of Epigenetics and Molecular Carcinogenesis, Division of Science Park, The University of Texas M. D. Anderson Cancer Center, Smithville, Texas, USA; g Laufer Center for Physical and Quantitative Biology, Stony Brook University, Stony Brook, New York, USA; University of Pittsburgh School of Medicine

**Keywords:** DNA replication, dUTPase, gammaherpesvirus, genomic stability, herpesviruses, latency, lytic replication, uracil-DNA glycosylase, viral pathogenesis, virus-host interactions

## Abstract

Misincorporation of uracil or spontaneous cytidine deamination is a common mutagenic insult to DNA. Herpesviruses encode a viral uracil-DNA glycosylase (vUNG) and a viral dUTPase (vDUT), each with enzymatic and nonenzymatic functions. However, the coordinated roles of these enzymatic activities in gammaherpesvirus pathogenesis and viral genomic stability have not been defined. In addition, potential compensation by the host UNG has not been examined *in vivo*. The genetic tractability of the murine gammaherpesvirus 68 (MHV68) system enabled us to delineate the contribution of host and viral factors that prevent uracilated DNA. Recombinant MHV68 lacking vUNG (ORF46.stop) was not further impaired for acute replication in the lungs of UNG^−/−^ mice compared to wild-type (WT) mice, indicating host UNG does not compensate for the absence of vUNG. Next, we investigated the separate and combinatorial consequences of mutating the catalytic residues of the vUNG (ORF46.CM) and vDUT (ORF54.CM). ORF46.CM was not impaired for replication, while ORF54.CM had a slight transient defect in replication in the lungs. However, disabling both vUNG and vDUT led to a significant defect in acute expansion in the lungs, followed by impaired establishment of latency in the splenic reservoir. Upon serial passage of the ORF46.CM/ORF54.CM mutant in either fibroblasts or the lungs of mice, we noted rapid loss of the nonessential yellow fluorescent protein (YFP) reporter gene from the viral genome, due to recombination at repetitive elements. Taken together, our data indicate that the vUNG and vDUT coordinate to promote viral genomic stability and enable viral expansion prior to colonization of latent reservoirs.

## Abstract (precis)

Unrepaired uracils in DNA can lead to mutations and compromise genomic stability. Herpesviruses have hijacked host processes of DNA repair and nucleotide metabolism by encoding a viral UNG that excises uracils and a viral dUTPase that initiates conversion of dUTP to dTTP. To better understand the impact of these processes on gammaherpesvirus pathogenesis, we examined the separate and collaborative roles of vUNG and vDUT upon MHV68 infection of mice. Simultaneous disruption of the enzymatic activities of both vUNG and vDUT led to a severe defect in acute replication and establishment of latency, while also revealing a novel, combinatorial function in promoting viral genomic stability. We propose that herpesviruses require these enzymatic processes to protect the viral genome from damage, possibly triggered by misincorporated uracil. This reveals a novel point of therapeutic intervention to potentially block viral replication and reduce the fitness of multiple herpesviruses.

## INTRODUCTION

Uracils in DNA are sources of mutations and lesions that impact genomic stability if not repaired properly (1). The degree of uracil misincorporation during DNA replication is moderated by the dUTP/dTTP ratio, while deamination of cytosine to uracil occurs at a rate of 60 to 500 events per cell per day (1–4). In addition, the apolipoprotein B mRNA-editing enzyme catalytic polypeptide-like 3 (APOBEC3) host DNA cytidine deaminases have been reported to drive mutations in herpes simplex virus 1 (HSV-1) and Epstein-Barr virus (EBV) (5). The herpesviruses encode homologs of host factors for DNA repair and nucleotide metabolism, including the enzymatically active viral uracil-DNA glycosylase (vUNG) and viral dUTPase (vDUT) (6). While it is generally believed that this family of DNA viruses evolved these host processes as a countermeasure to the mutagenic insult of uracilated DNA, the consequence of their enzymatic functions for herpesvirus pathogenesis and viral genomic stability has not been well characterized.

Uracils in DNA are recognized and excised by members of the uracil-DNA glycosylase (UDG) family, which includes uracil-*N*-glycosylase 1/2 (UNG1/2), single-strand selective monofunctional uracil-DNA glycosylase (SMUG1), thymine-DNA glycosylase (TDG), and methyl-CpG-binding domain 4 (MBD4). UNG2 is the nuclear isoform that recognizes a variety of substrates (ssU, U:G, U:A), wherein excision of uracil leads to base excision repair (BER) of the abasic site to maintain fidelity in most cell types (7). In germinal center B cells that are undergoing immunoglobulin gene rearrangement and mutation, activation-induced cytidine deaminase (AID) initiates cytidine deamination that is followed by UNG2 to mediate error-prone DNA repair (8). All herpesviruses encode a homolog of uracil-DNA glycosylase that is closely related to host UNG2 (vUNG); those examined thus far are enzymatically active (6, 9, 10). We recently reported that the vUNG of murine gammaherpesvirus 68 (MHV68 [formally murid herpesvirus 4]) encoded by open reading frame 46 (ORF46) can substitute for the host UNG2 to mediate immunoglobulin gene isotype class switching in B cells (10).

The replication of herpesvirus mutants with disruptions in the vUNG ORF has been examined in both cell culture and animal models. The vUNG of HSV-1 (UL2) is essential for replication and spread in both the peripheral and central nervous systems (CNS) (11). However, another alphaherpesvirus, varicella zoster virus (VZV), does not require viral or host UNG activity for DNA replication in human melanoma cells (12). Human cytomegalovirus (HCMV) vUNG (UL114) promotes DNA synthesis and viral replication in human embryonic lung cells (13) and human fibroblast cells with low host UNG expression (14, 15). Reactivation from EBV^+^ nasopharyngeal carcinoma cells in the absence of EBV vUNG (BKRF3) leads to a reduction in viral DNA synthesis (16). We previously reported that while MHV68 vUNG (ORF46) is not essential for replication in murine fibroblast cells, the protein is required for acute replication and expansion in the lungs and downstream colonization of the latency reservoirs of infected mice (10).

Moreover, herpesvirus vUNGs interact with viral DNA replication factors. HSV-1 vUNG interacts with viral DNA polymerase catalytic subunit (UL30) (17). HCMV vUNG was shown to interact with viral DNA polymerase (UL54) and viral DNA polymerase processivity factor (UL44) (13, 18, 19). EBV vUNG associates with the viral DNA polymerase (BALF5), viral DNA polymerase processivity factor (BMRF1), and the latent-lytic switch replication and transcription activator, RTA (9). Thus, the mechanistic basis for vUNG promotion of DNA replication may involve uracil excision and potential scaffolding interactions with other viral proteins.

High levels of cellular dUTP increase uracil misincorporation in DNA. Host dUTPase cleaves dUTP to yield dUMP for thymidine synthesis, effectively reducing the dUTP pool to limit uracil misincorporation (2). Herpesviruses of the alphaherpesvirus (HSV-1, VZV) and gammaherpesvirus (EBV, Kaposi’s sarcoma-associated herpesvirus [KSHV], MHV68) subfamilies encode enzymatically active viral dUTPases (6, 20, 21), but homologs in HCMV are not active (22). HSV-1 vDUT (UL50) is the most studied among all herpesvirus dUTPases. vDUT is required for HSV-1 replication and pathogenicity in mice (23, 24). Loss of HSV-1 vDUT results in increased loss of β-galactosidase (β-Gal) reporter activity from the recombinant virus in murine fibroblasts (25). Furthermore, the enzymatic activity of HSV-1 vDUT is modulated by Us3-mediated phosphorylation and plays a critical role in HSV-1 replication in the CNS (24, 26, 27). Low HSV-1 vDUT enzymatic activity results in a higher frequency of mutations in viral DNA, which is rescued by host dUTPase expression (27). However, a nonenzymatic function of HSV-1 vDUT is also required for efficient replication in ocular and vaginal tissues (24). Murine gammaherpesvirus 68 fails to establish persistent infection in the absence of vDUT (ORF54) due to an anti-interferon function that is distinct from its enzymatic activities (21); KSHV vDUT (ORF54) has a similar anti-interferon function (21). EBV vDUT (BLLF3) is secreted by infected cells to activate Toll-like receptors and modulate immune responses (28). Thus, as found for the herpesvirus vUNGs, vDUTs have functions distinct from their enzymatic namesakes and require careful mutagenic strategies to dissect their roles in pathogenesis.

Similar to what was found for other herpesvirus proteins such as the viral ribonucleotide reductase (RNR) subunits and thymidine kinase (TK), which are involved in nucleotide metabolism, vDUT and vUNG are dispensable for propagation under typical cell culture conditions (29). However, it is clear that these accessory proteins influence pathogenesis and are required for viral replication in quiescent, restrictive cells *in vivo* (30–33). In this study, we utilized MHV68 infection in its natural host to address critical questions regarding the contribution of the vUNG and vDUT to herpesvirus replication and pathogenesis. Since herpesviruses require vUNG to replicate efficiently in cells or tissues with limited host UNG activities (10, 11, 15, 16), it is believed that the vUNG compensates for the lack of the host UNG. This led us to investigate whether infection in UNG^−/−^ mice would reveal novel or exacerbated phenotypes for the vUNG mutant. In addition, to dissect enzymatic from potential scaffolding functions of MHV68 vUNG and vDUT, we generated and characterized single and combinatorial enzymatic mutants of ORF46 and ORF54 *in vivo*. Our pathogenesis studies demonstrated that the host UNG does not compensate for the viral UNG, and the vUNG has important nonenzymatic functions. In addition, neither the vUNG or vDUT single mutant virus exhibited a severe replication defect in mice, indicating potential molecular compensation between these two enzymes. Thus, we generated a combinatorial mutant virus with inactive forms of both vUNG and vDUT. Consistent with our hypothesis, we observed greater defects in viral replication in the lung and dissemination to the spleen with the double mutant MHV68. Following serial passage *in vitro* and *in vivo*, there was a striking loss of a nonessential yellow fluorescent protein (YFP) reporter gene via recombination. Taken together, our data demonstrate that the combined enzymatic activities of vUNG and vDUT are required for viral genomic stability and promotion of viral replication and pathogenesis in mice.

## RESULTS

### Absence of host UNG does not impact MHV68 pathogenesis in mice.

**FIG 1:**
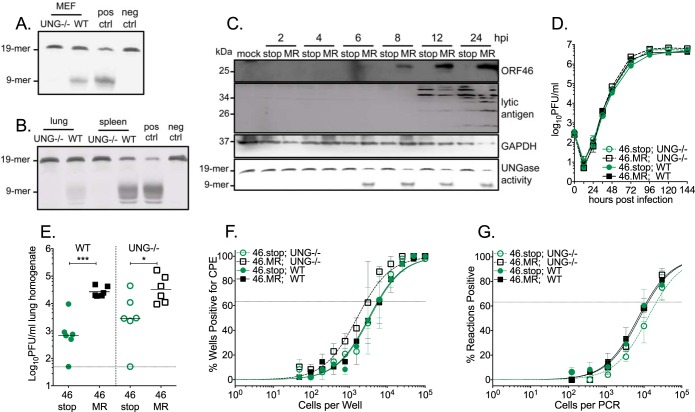
The function of the viral UNG encoded by ORF46 is not compensated by host UNG during pathogenesis. (A) The UNGase assay was performed with lysates prepared from UNG^−/−^ or WT MEFs. UNGase activity was measured in lysates by the generation of a 9-mer cleavage product upon incubation with a 19-mer oligonucleotide containing a single uracil. (B) UNGase assay on lung and spleen tissue from UNG^−/−^ or WT mice. (C) Time course of ORF46 expression and UNGase activity. UNG^−/−^ MEFs were infected with recombinant MHV68 with a stop codon disruption in ORF46 (46.stop [stop lanes]) or with the marker rescue virus of ORF46.stop (46.MR [MR lanes]). The indicated viral and host proteins were detected by immunoblotting. (D) Multistep growth curve in primary UNG^−/−^ and WT MEFs with the indicated viruses (MOI of 0.01). (E) Acute replication in the lungs of UNG^−/−^ or WT C57BL/6 mice infected by the intranasal route with 1,000 PFU of the indicated viruses (M3Luc) at 9 dpi. Each symbol represents the titer per milliliter of lung homogenate in an individual mouse. The line indicates the geometric mean titer. The dashed line depicts the limit of detection at 50 PFU/ml. (F and G) UNG^−/−^ mice or WT C57BL/6 mice were infected by the intraperitoneal route with 1,000 PFU of the indicated viruses. (F) Reactivation frequency of splenocytes 16 dpi was examined by limiting dilution assay. Data were generated from two independent experiments with 3 to 6 mice per group (G) Frequency of MHV68 genome-positive splenocytes 42 dpi was determined by limiting dilution PCR. Data were generated from four replicates per condition. For the limiting dilution analyses, curve fit lines were determined by nonlinear regression analysis. Using Poisson analysis, the intersection of the nonlinear regression curves with the dashed line at 63.2% was used to determine the frequency of cells that were either positive for the viral genome or reactivating virus. For panel E, significance was determined by two-way unpaired *t* test on infected animals: *, *P* < 0.05; ***, *P* < 0.001.

To better distinguish the contribution of the viral UNG from the host UNG in the context of infection, we examined virus replication and pathogenesis in mice lacking mitochondrial and nuclear isoforms of host UNG1/2 (UNG^−/−^) (34). To validate the absence of host UNG activity in UNG^−/−^ mice, we examined the cleavage of a single-stranded uracil-containing oligonucleotide upon incubation with lysates of primary murine embryonic fibroblasts (MEFs) and other tissues from UNG^−/−^ and wild-type (WT) mice. We observed no oligonucleotide cleavage in samples from UNG^−/−^ mice (Fig. 1A and B). We next examined the expression and activity of the vUNG upon the infection of host UNG^−/−^ MEFs with the ORF46.stop virus (46.stop) and the ORF46.stop marker rescue (MR) virus (46.MR). vUNG was detected by immunoblotting at 6 h postinfection (hpi), and levels increased with time in the 46.MR-infected UNG^−/−^ MEFs (Fig. 1C). UNG activity was also detected upon infection with 46.MR, but not 46.stop (Fig. 1C). We next examined replication kinetics of 46.stop and 46.MR in both UNG^−/−^ and WT MEFs. 46.stop replication was indistinguishable from that of 46.MR, regardless of the absence (UNG^−/−^) or presence (WT) of host UNG (Fig. 1D). These data suggest that uracil glycosylase activity, whether derived from the host or the virus, is not required for MHV68 replication in fibroblasts.

Our laboratory previously observed that MHV68 had tissue-specific defects in the absence of the vUNG (ORF46) *in vivo* (10). The ORF46.stop mutant was impaired for replication in the lungs, which exhibit low host uracil-DNA glycosylase activity, while there was no phenotype in splenocytes, which exhibit higher host UNG activity. We hypothesized that the host UNG might compensate for the absence of the viral UNG in some tissues. Upon intranasal infection of WT mice, 46.stop had an approximate 1-log defect in acute replication in the lungs 9 days postinfection (dpi) compared to 46.MR (Fig. 1E). 46.stop replication was reduced by a similar level in the UNG^−/−^ mice, suggesting that the host UNG does not contribute to replication in the lungs. We next examined the role of the host UNG in MHV68 colonization of the spleen following intraperitoneal inoculation, an experimental design that bypasses the acute replication defect of 46.stop in the lung. The absence of host UNG in the splenocytes did not impact the frequency of reactivation from latency upon explant and coculture of MEFs with splenocytes from mice infected with 46.stop or 46.MR 16 dpi (Fig. 1F and Table 1). Furthermore, based on the frequency of genome-positive splenocytes, long-term latency establishment was not impacted by the lack of viral and host UNGs (Fig. 1G and Table 2). Taken together, these results indicate that while the vUNG protein is required for acute replication, the host UNG does not influence acute replication in the lung or splenic latency of WT or vUNG-null MHV68 in the infected animal.

**TABLE 1:** Frequency of cell populations reactivating latent virus in C57BL/6 mice 16 dpi

Virus^*a*^	Route of infection^*b*^	Mouse	Organ^*c*^	Total no. of cells harvested^*d*^	Frequency of cells reactivating latent virus^*e*^	Total no. of cells reactivating latent virus^*f*^
ORF46.stop	i.n.	WT	Spleen	1.8 × 10^8^	BLD^*g*^^,^^*h*^	BLD^*h*^
ORF46.stop MR	i.n.	WT	Spleen	3.1 × 10^8^	3,191^*g*^	9.6 × 10^4^
ORF46.CM	i.n.	WT	Spleen	2.4 × 10^8^	7,223	3.3 × 10^4^
ORF46.CM MR	i.n.	WT	Spleen	2.2 × 10^8^	2,361	9.2 × 10^4^
ORF54.CM	i.n.	WT	Spleen	1.1 × 10^8^	90,490	1.2 × 10^3^
ORF54.CM MR	i.n.	WT	Spleen	1.4 × 10^8^	6,401	2.1 × 10^4^
46.CM/54.CM	i.n.	WT	Spleen	9.8 × 10^7^	545,918	1.8 × 10^2^
46.CM/54.CM MR	i.n.	WT	Spleen	1.4 × 10^8^	4,413	3.1 × 10^4^
ORF46.CM	i.p.	WT	Spleen	2.7 × 10^8^	3,235	8.4 × 10^4^
ORF46.CM MR	i.p.	WT	Spleen	2.9 × 10^8^	3,875	7.4 × 10^4^
ORF54.CM	i.p.	WT	Spleen	2.3 × 10^8^	17,668	1.3 × 10^4^
ORF54.CM MR	i.p.	WT	Spleen	2.3 × 10^8^	5,941	3.8 × 10^4^
46.CM/54.CM	i.p.	WT	Spleen	2.4 × 10^8^	7,055	3.4 × 10^4^
46.CM/54.CM MR	i.p.	WT	Spleen	2.3 × 10^8^	15,531	1.5 × 10^4^
ORF46.stop	i.p.	UNG^−/−^	Spleen	2.5 × 10^8^	6,021	4.2 × 10^4^
	i.p.	WT	Spleen	2.1 × 10^8^	7,297	2.9 × 10^4^
ORF46.stop MR	i.p.	UNG^−/−^	Spleen	3.1 × 10^8^	3,255	9.6 × 10^4^
	i.p.	WT	Spleen	2.0 × 10^8^	6,765	3.0 × 10^4^
ORF46.CM	i.p.	WT	PECs	1.9 × 10^7^	1,724	1.1 × 10^4^
ORF46.CM MR	i.p.	WT	PECs	3.4 × 10^7^	1,495	2.3 × 10^4^
ORF54.CM	i.p.	WT	PECs	1.5 × 10^7^	835	1.8 × 10^4^
ORF54.CM MR	i.p.	WT	PECs	1.2 × 10^7^	278	4.2 × 10^4^
46.CM/54.CM	i.p.	WT	PECs	8.9 × 10^6^	804	1.1 × 10^4^
46.CM/54.CM MR	i.p.	WT	PECs	1.2 × 10^7^	756	1.6 × 10^4^

a Infection with recombinant MHV68 viruses.

b i.n., intranasal; i.p., intraperitoneal.

c Organ harvested for limiting dilution analysis.

d The total number of cells harvested per mouse was determined by the mean of pooled from three to six mice per experiment unless indicated elsewhere.

e Data represent frequencies of the indicated cells (1 in *x*) that reactivated from latency based on Poisson distribution analysis. The frequency data were determined from the mean of two to five independent experiments with cells from the indicated organs; organs were pooled from three to six mice per experiment unless indicated elsewhere.

f The total number of cells reactivating latent virus per mouse was extrapolated using the frequency value generated from the limiting dilution analysis together with the total number of splenocytes or PECs harvested.

g The frequency data were determined from one experiment with splenocytes pooled from four to five mice.

h BLD, below the limit of detection.

**TABLE 2:** Frequencies of cells harboring viral genomes in C57BL/6 mice

Virus^*a*^	Route of infection^*b*^	Mouse	Organ^*c*^	No. of dpi	Total no. of cells harvested^*d*^	Frequency of genome-positive cells^*e*^	Total no. of cells positive for latent virus^*f*^
ORF46.stop	i.n.	WT	Spleen	16	1.8 × 10^8^	BLD^*g*^^,^^*h*^	BLD^*h*^
ORF46.stop MR	i.n.	WT	Spleen	16	3.1 × 10^8^	302^*g*^	1.0 × 10^6^
ORF46.CM	i.n.	WT	Spleen	16	2.4 × 10^8^	578	4.1 × 10^5^
ORF46.CM MR	i.n.	WT	Spleen	16	2.2 × 10^8^	328	6.6 × 10^5^
ORF54.CM	i.n.	WT	Spleen	16	1.1 × 10^8^	4,197	2.5 × 10^4^
ORF54.CM MR	i.n.	WT	Spleen	16	1.4 × 10^8^	437	3.1 × 10^5^
46.CM/54.CM	i.n.	WT	Spleen	16	9.8 × 10^7^	24,346	4.0 × 10^3^
46.CM/54.CM MR	i.n.	WT	Spleen	16	1.4 × 10^8^	266	5.2 × 10^5^
ORF46.CM	i.n.	WT	Spleen	42	1.6 × 10^8^	7,837	2.0 × 10^4^
ORF46.CM MR	i.n.	WT	Spleen	42	1.4 × 10^8^	2,458	5.8 × 10^4^
ORF54.CM	i.n.	WT	Spleen	42	1.1 × 10^8^	2,258^*g*^	4.6 × 10^4^
ORF54.CM MR	i.n.	WT	Spleen	42	1.3 × 10^8^	1,840^*g*^	7.0 × 10^4^
46.CM/54.CM	i.n.	WT	Spleen	42	1.5 × 10^8^	18,798	7.8 × 10^3^
46.CM/54.CM MR	i.n.	WT	Spleen	42	1.5 × 10^8^	6,801	2.1 × 10^4^
ORF46.CM	i.p.	WT	Spleen	16	2.7 × 10^8^	154	1.8 × 10^6^
ORF46.CM MR	i.p.	WT	Spleen	16	2.9 × 10^8^	248	1.2 × 10^6^
ORF54.CM	i.p.	WT	Spleen	16	2.3 × 10^8^	393	5.7 × 10^5^
ORF54.CM MR	i.p.	WT	Spleen	16	2.3 × 10^8^	226	1.0 × 10^6^
46.CM/54.CM	i.p.	WT	Spleen	16	2.4 × 10^8^	351	6.8 × 10^5^
46.CM/54.CM MR	i.p.	WT	Spleen	16	2.3 × 10^8^	294	7.8 × 10^5^
ORF46.CM	i.p.	WT	PECs	16	1.9 × 10^7^	203	9.6 × 10^4^
ORF46.CM MR	i.p.	WT	PECs	16	3.4 × 10^7^	131	2.6 × 10^5^
ORF54.CM	i.p.	WT	PECs	16	1.5 × 10^7^	146	1.0 × 10^5^
ORF54.CM MR	i.p.	WT	PECs	16	1.2 × 10^7^	86	1.4 × 10^5^
46.CM/54.CM	i.p.	WT	PECs	16	8.9 × 10^6^	184	4.9 × 10^4^
46.CM/54.CM MR	i.p.	WT	PECs	16	1.2 × 10^7^	85	1.4 × 10^5^
ORF46.stop	i.p.	UNG^−/−^	Spleen	42	7.2 × 10^7^	24,804^*i*^	2.9 × 10^3^
	i.p.	WT	Spleen	42	6.5 × 10^7^	22,078^*i*^	2.9 × 10^3^
ORF46.stop MR	i.p.	UNG^−/−^	Spleen	42	7.0 × 10^7^	13,445^*i*^	5.2 × 10^3^
	i.p.	WT	Spleen	42	5.7 × 10^7^	13,060^*i*^	4.4 × 10^3^
ORF46.CM	i.p.	WT	Spleen	42	1.5 × 10^8^	4,438	3.5 × 10^4^
ORF46.CM MR	i.p.	WT	Spleen	42	1.9 × 10^8^	3,219	6.0 × 10^4^
46.CM/54.CM	i.p.	WT	Spleen	42	1.5 × 10^8^	2,579	5.8 × 10^4^
46.CM/54.CM MR	i.p.	WT	Spleen	42	1.3 × 10^8^	1,730	7.6 × 10^4^

a Infection with recombinant MHV68 viruses.

b i.n., intranasal; i.p., intraperitoneal.

c Organ harvested for limiting dilution analysis.

d The total number of cells harvested per mouse was determined by the mean of pooled cells from three to six mice per experiment unless indicated elsewhere.

e Data represent frequencies of the indicated cells (1 in *x*) that harbored viral genome based on Poisson distribution analysis. The frequency data were determined from the mean of two to five independent experiments with cells from the indicated organs; organs were pooled from three to six mice per experiment unless indicated elsewhere.

f The total number of genome-positive cells per mouse was extrapolated using the frequency value generated from the limiting dilution analysis together with the total number of splenocytes or PECs harvested.

g The frequency data were determined from one experiment with splenocytes pooled from four to five mice.

h BLD, below the limit of detection.

i The frequency data were determined from the mean of four to five mice with the splenocytes harvested.

### Enzymatic activity of MHV68 vUNG is not required for pathogenesis.

**FIG 2:**
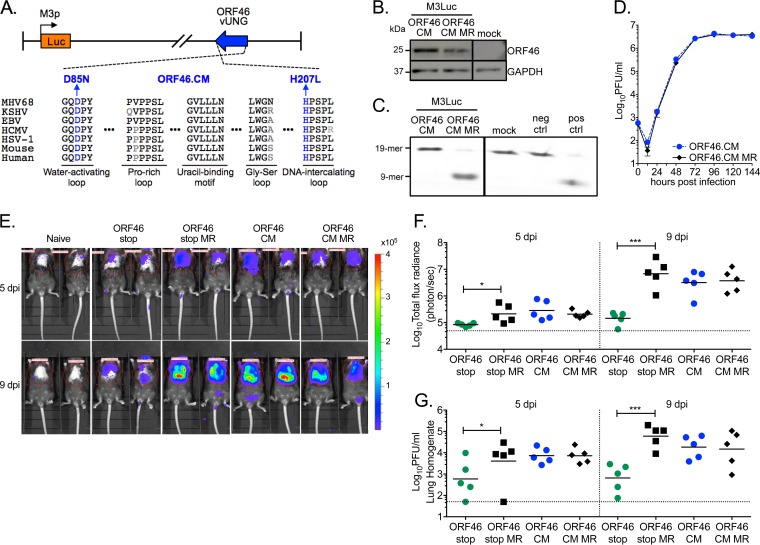
MHV68 lacking the enzymatic activity of vUNG has no replication defect in cell culture or in mouse lungs. (A) Schematic of the MHV68 vUNG catalytic mutant virus (ORF46.CM) with the luciferase reporter gene under the control of the M3 lytic promoter (M3Luc). Alignment of the conserved enzymatic motifs (black amino acids) and the location of the two mutations in ORF46.CM (blue amino acids) are presented below. (B) Immunoblot validation of mutant ORF46 expression in infected UNG^−/−^ MEFs. (C) Lack of UNGase activity in lysates of UNG^−/−^ cells infected with ORF46.CM. (D) Multistep growth curve in primary WT MEFs with the indicated viruses (MOI of 0.01). (E to G) WT C57BL/6 mice were infected by the intranasal route with 1,000 PFU of the indicated viruses. (E) Representative pictures of mock-infected or MHV68-infected mice upon *in vivo* imaging of chemiluminescence in the thorax at 5 and 9 dpi. The scale bar shows photons s^−1 ^cm^−2 ^sr^−1^. (F) Each symbol represents the total flux radiance within the region of interest for an individual mouse. The dashed line depicts the limit of detection at 5 × 10^4^ photons s^−1^. (G) Acute replication in lungs imaged in panel F determined by plaque assay. Each symbol represents the titer per milliliter of lung homogenate in an individual mouse. The line indicates the geometric mean titer. The dashed line depicts the limit of detection at 50 PFU/ml. For panels F and G, significance was determined by two-way unpaired *t* test on infected animals: *, *P* < 0.05; ***, *P* < 0.001.

To investigate whether the enzymatic activity of vUNG facilitates acute replication in the murine lung, we generated a recombinant MHV68 (ORF46.CM) with two amino acid changes in motifs that are essential for catalytic activity and conserved across all herpesvirus and host UNGs. We mutated an aspartic acid to asparagine (D85N) in the water-activating loop and a histidine to leucine (H207L) within the DNA intercalating loop (Fig. 2A). The catalytic vUNG mutant encoded by ORF46.CM was expressed at a similar level as WT vUNG (Fig. 2B), but demonstrated no glycosylase activity in UNG^−/−^ MEFs (Fig. 2C). Consistent with ORF46.stop, ORF46.CM had no replication defect compared to ORF46.CM MR virus in a multistep growth curve in WT MEFs (Fig. 2D).

To track MHV68 acute replication via *in vivo* imaging, ORF46 mutants and their MR controls were generated in a recombinant virus that carries the luciferase reporter gene under the control of the M3 lytic promoter (M3pLuc) (Fig. 2A) (35). We infected WT mice with ORF46.stop or ORF46.CM and their respective MR controls by the intranasal route and imaged replication by chemiluminescence 5 and 9 dpi. Mice infected with the MR control viruses exhibited a strong production of luciferase in the thorax at 5 dpi compared to uninfected mice, and luciferase activity was substantially increased by 9 dpi (Fig. 2E and F). Mice infected with ORF46.stop had significantly less chemiluminescence signal in the thorax than those infected with ORF46.stop MR on both 5 and 9 dpi (Fig. 2E and F). In contrast, the mice infected with the ORF46.CM virus had luciferase levels comparable to those of mice infected with ORF46.CM MR (Fig. 2E and F). Virus titer determined by plaque assay was concordant with the luciferase activity determined for all viruses (Fig. 2G). The infection of UNG^−/−^ mice did not reveal a replication defect for ORF46.CM (see Fig. S1A in the supplemental material). Our analysis of ORF46.CM demonstrated that the lack of the uracil glycosylase activity of vUNG does not impact viral replication in the lungs, in sharp contrast to the significant defect upon infection with ORF46.stop.

10.1128/mBio.01831-18.2 FIG S1 MHV68 lacking the enzymatic activity of the vUNG has no defect in lytic replication, establishing latency in PECs or long-term latency in spleens. (A) UNG^−/−^ and WT mice were infected by intranasal route with 1,000 PFU of the indicated viruses. Acute replication in the lungs 9 dpi was determined by plaque assay. The line indicates the geometric mean titer. The dashed line depicts the limit of detection at 50 PFU/ml of lung homogenate. WT C57BL/6 mice were infected by either the intraperitoneal (IP [B to E]) or intranasal (IN [F to G]) route with 1,000 PFU of the indicated viruses. (B) Frequency of PECs harboring latent genomes at 16 dpi. (C) Frequency of PECs capable of reactivation from latency upon explant at 16 dpi. (D and F) Weights of spleens harvested at 42 dpi. (E and G) Frequency of splenocytes harboring latent genomes at 42 dpi. For the limiting dilution analyses, curve fit lines were determined by nonlinear regression analysis. Using Poisson analysis, the intersection of the nonlinear regression curves with the dashed line at 63.2% was used to determine the frequency of cells that were either positive for the viral genome or reactivating virus. For panels A, D, and F, each symbol represents an individual mouse. For panels B to C, E, and G, the data are generated from two independent experiments with 3 to 6 mice per group. Download FIG S1, EPS file, 0.3 MB. Copyright © 2018 Dong et al. 2018 Dong et al. This content is distributed under the terms of the Creative Commons Attribution 4.0 International license.

**FIG 3:**
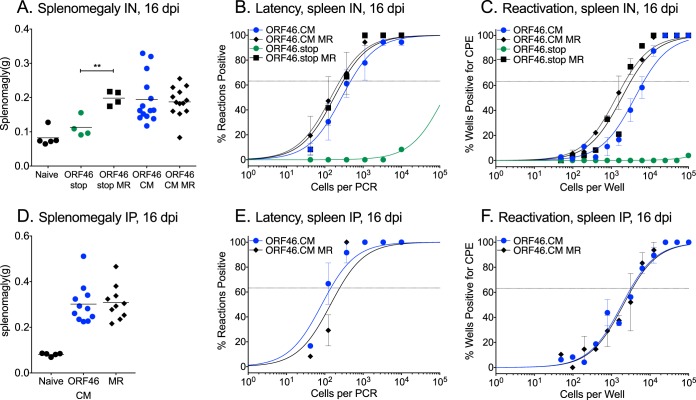
MHV68 lacking the enzymatic activity of vUNG has no defect in the establishment of latency or reactivation from latency in mice. WT C57BL/6 mice were infected by either the intranasal (IN [A to C]) or intraperitoneal (IP [D to F]) route with 1,000 PFU of the indicated viruses. (A and D) Weights of spleens from uninfected, naive mice or infected mice at 16 dpi. (B and E) Frequency of splenocytes harboring latent genomes at 16 dpi. (C and F) Frequency of splenocytes capable of reactivation from latency upon explant at 16 dpi. For the limiting dilution analyses, curve fit lines were determined by nonlinear regression analysis. Using Poisson analysis, the intersection of the nonlinear regression curves with the dashed line at 63.2% was used to determine the frequency of cells that were either positive for the viral genome or reactivating virus. For panels A to C, data are generated from three independent experiments with 3 to 6 mice per group. For panels D to H, data are generated from two experiments with 3 to 6 mice per group. Error bars indicate standard errors of the mean (SEM). For panel A, ** indicates *P* < 0.01 by two-way unpaired *t* test.

Next, the role of the enzymatic activity of the vUNG in splenic latency was examined upon infection of mice with ORF46.CM in parallel with ORF46.stop. As previously reported, the absence of ORF46 led to a severe defect in splenomegaly (Fig. 3A), latency establishment (Fig. 3B), and reactivation from latency (Fig. 3C) 16 days after intranasal infection. However, mice infected with ORF46.CM exhibited robust splenomegaly (Fig. 3A), established latency (Fig. 3B and Table 2), and reactivated from latency (Fig. 3C and Table 1) at levels indistinguishable from those of mice infected with ORF46.CM MR. Furthermore, upon intraperitoneal infection, mice infected with ORF46.CM exhibited the same degree of splenomegaly (Fig. 3D) and frequencies of latency and reactivation that were indistinguishable from those of their MR-infected counterparts (Fig. 3E and F and Table 1 and Table 2). There were also no defects in latency establishment or reactivation from latency in the peritoneal exudate cells (PECs) of mice infected with ORF46.CM compared to ORF46.CM MR (Fig. S1B and C). Regardless of the route of infection, the mice infected with ORF46.CM were indistinguishable from mice infected with MR based on the degree of splenomegaly and frequency of splenic latency 42 dpi (Fig. S1D to G). Taken together, the absence of vUNG is detrimental to replication in the lungs, in turn delaying the establishment of latency in the spleen. However, we observed no replication or latency defect for ORF46.CM, indicating that the vUNG enzymatic activity is dispensable for most aspects of MHV68 pathogenesis in mice. These data suggest that vUNG provides an enzymatic-independent role supporting viral replication and expansion during acute infection.

### Enzymatic activity of MHV68 vDUT has a transient impact on viral replication in the lung and promotes latency establishment in the spleen.

**FIG 4:**
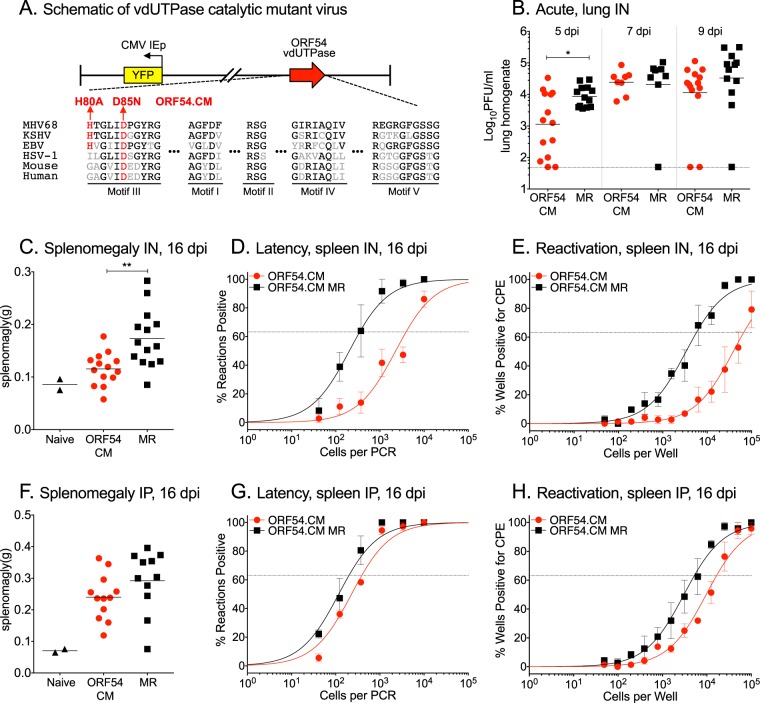
MHV68 lacking the enzymatic activity of the vDUT encoded by ORF54 leads to a transient decrease in acute replication and a reduction in splenic latency at 16 dpi. (A) Schematic of the MHV68 vDUT catalytic mutant virus (ORF54.CM) with the H2BYFP reporter gene located between ORF27 and ORF29b. Alignment of the conserved enzymatic motifs (black amino acids) and the locations of the two mutations in ORF54.CM (red amino acids) are below. WT C57BL/6 mice were infected by either the intranasal (IN [B to E]) or intraperitoneal (IP [F to H]) route with 1,000 PFU of the indicated viruses. (B) Acute replication in the lungs at 5, 7, and 9 dpi. Each symbol represents the titer per milliliter of lung homogenate in an individual mouse. The line indicates the geometric mean titer. The dashed line depicts the limit of detection at 50 PFU/ml. (C and F) Weights of spleens from uninfected, naive mice or infected mice at 16 dpi. (D and G) Frequency of splenocytes harboring latent genomes at 16 dpi. (E and H) Frequency of splenocytes capable of reactivation from latency upon explant at 16 dpi. For the limiting dilution analyses, curve fit lines were determined by nonlinear regression analysis. Using Poisson analysis, the intersection of the nonlinear regression curves with the dashed line at 63.2% was used to determine the frequency of cells that were either positive for the viral genome or reactivating virus. For panels C to H, data were generated from three independent experiments, with 3 to 6 mice per group. Error bars indicate SEM. For panels B and C, significance was determined by two-way unpaired *t* test on infected animals: *, *P* < 0.05; **, *P* < 0.01.

The vDUTs of MHV68 (ORF54) and other herpesviruses dephosphorylate dUTP (6, 20, 21), an enzymatic activity shared with host dUTPase that promotes the metabolism of dTTP. Thus, vDUT is thought to reduce the dUTP/dTTP ratio and thereby limit misincorporation of uracils in the viral genome. To investigate the role of vDUT enzymatic activity during MHV68 pathogenesis, we generated an ORF54 enzymatic mutant virus (ORF54.CM) with two amino acid changes in enzymatic motif III (H80A and D85N) that are also conserved across gammaherpesvirus vDUTs (Fig. 4A), as previously described (21). Upon intranasal infection of WT mice, ORF54.CM exhibited a transient, 1-log replication defect in the lung at 5 dpi, yet replicated to comparable levels to the ORF54.CM MR virus at 7 and 9 dpi (Fig. 4B). Splenomegaly at 16 dpi was reduced in the mice infected with ORF54.CM compared to MR virus (Fig. 4C). A 1-log defect in latency establishment was observed for splenocytes infected with ORF54.CM compared to those infected with ORF54.CM MR (Fig. 4D and Table 2), consistent with the 1-log defect in reactivation from latency (Fig. 4E and Table 1). By 42 dpi, ORF54.CM was detected at similar frequencies to MR (see Fig. S2A and B in the supplemental material). In contrast, no substantial differences in spleen weight, latency establishment, or reactivation from splenic latency were observed following intraperitoneal infection with ORF54.CM (Fig. 4F to H). Analysis of latency in the PECs at 16 dpi did not identify a phenotype for ORF54.CM (Fig. S2C and D). In summary, the dUTPase activity of ORF54 promotes early replication during acute infection. As observed for other MHV68 mutants impaired for acute replication (10, 30–33, 36), ORF54.CM was compromised in latency establishment at 16 dpi, but reached levels comparable to those of the MR virus by 42 dpi. These phenotypes were not observed for a similar ORF54.CM mutant in BALB/c mice (21). Our data suggest that the dUTPase activity of ORF54 has a tissue-specific role in gammaherpesvirus pathogenesis in C57BL/6 mice.

10.1128/mBio.01831-18.3 FIG S2 vDUT enzymatic function is not essential for viral latency establishment upon intraperitoneal infection or long-term latency. C57BL/6 mice were infected by the intranasal (IN [A and B]) or intraperitoneal (IP [C and D]) route with 1,000 PFU of the indicated viruses. (A) Weights of spleens harvested at 42 dpi. (B) Frequency of splenocytes harboring latent genomes at 42 dpi. (C) Frequency of PECs harboring latent genomes at 16 dpi. (D) Frequency of PECs capable of reactivation from latency upon explant at 16 dpi. For the limiting dilution analyses, curve fit lines were determined by nonlinear regression analysis. Using Poisson analysis, the intersection of the nonlinear regression curves with the dashed line at 63.2% was used to determine the frequency of cells that were either positive for the viral genome or reactivating virus. For panel A, each symbol represents an individual mouse. For panel B, the data were generated with 3 to 6 mice per group. For panels C and D, the data were generated from three independent experiments with 3 to 6 mice per group. Download FIG S2, EPS file, 0.2 MB. Copyright © 2018 Dong et al. 2018 Dong et al. This content is distributed under the terms of the Creative Commons Attribution 4.0 International license.

### The enzymatic activities of the vUNG and vDUT synergize to promote pathogenesis in mice.

**FIG 5:**
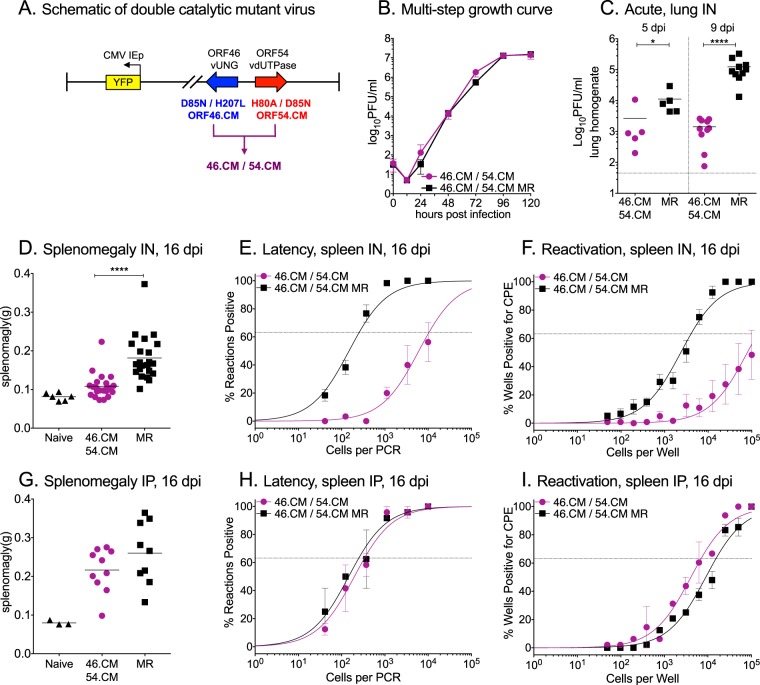
Loss of the enzymatic activity of both vUNG and vDUT does not impact MHV68 replication in cell culture, but reduces viral replication in the lung and seeding of the latency reservoir in the spleen after intranasal inoculation of mice. (A) Schematic of the combinatorial vUNG and vDUT catalytic mutant virus (46.CM/54.CM) with the H2BYFP reporter gene located between ORF27 and ORF29b. (B) Multistep growth curve in primary WT MEFs with the indicated viruses (MOI of 0.01). WT C57BL/6 mice were infected by either the intranasal (IN [C to F]) or intraperitoneal (IP [G to I]) route with 1,000 PFU of the indicated viruses. (C) Acute replication in the lungs at 5 and 9 dpi. Each symbol represents the titer per milliliter of lung homogenate in an individual mouse. The line indicates the geometric mean titer. The dashed line depicts the limit of detection at 50 PFU/ml. (D and G) Weights of spleens from uninfected, naive mice or infected mice at 16 dpi. (E and H) Frequency of splenocytes harboring latent genomes at 16 dpi. (F and I) Frequency of splenocytes capable of reactivation from latency upon explant at 16 dpi. For the limiting dilution analyses, curve fit lines were determined by nonlinear regression analysis. Using Poisson analysis, the intersection of the nonlinear regression curves with the dashed line at 63.2% was used to determine the frequency of cells that were either positive for the viral genome or reactivating virus. For panels D to F, data are generated from five independent experiments with 3 to 6 mice per group. For panels G to I, data are generated from two independent experiments with 3 to 6 mice per group. Error bars indicate SEM. For panels C and D, significance was determined by two-way unpaired *t* test on infected animals: *, *P* < 0.05; ****, *P* < 0.0001.

Thus far, we observed that the single vUNG enzymatic mutant (ORF46.CM) had no defect in replication (Fig. 2), while the single vDUT mutant (ORF54.CM) had only a transient defect (Fig. 4). We reasoned that the activity of the vDUT maintains a lower dUTP/dTTP ratio and would thereby prevent uracil misincorporation and obviate a phenotype for the single vUNG enzymatic mutant. On the other hand, excision of misincorporated uracils by vUNG would mask the impact of a higher dUTP/dTTP ratio in the absence of the vDUT enzymatic activity. This led us to examine the potential synergy of these two enzymes by generating a combinatorial mutant virus with both of the enzymatic sites mutated in ORF46 and ORF54 (46.CM/54.CM) (Fig. 5A). We verified that each gene was expressed at similar levels in both the mutant- and MR-infected cells without impacting transcript levels of neighboring genes (see Fig. S3A to C in the supplemental material). The enzymatic activity of both vUNG and vDUT was lost in the 46.CM/54.CM-infected cells (Fig. S3D and E). We determined that the combinatorial 46.CM/54.CM mutant virus had no replication defect in WT MEFs (Fig. 5B). However, following intranasal infection of mice, the 46.CM/54.CM virus exhibited a significant replication defect in the lungs at 5 dpi. Moreover, replication of 46.CM/54.CM was further impaired by nearly 2 logs at 9 dpi (Fig. 5C). This contrasted sharply with the phenotype of the single mutants during acute replication. A similar replication defect of 46.CM/54.CM was observed in UNG^−/−^ mice (Fig. S3F).

10.1128/mBio.01831-18.4 FIG S3 Loss of both vUNG and vDUT activities does not impact replication in cell culture, yet reduces viral replication in the lung. (A) Immunoblot of mutant ORF46 expression in UNG^−/−^ MEFs. (B) Fibroblast cells were transduced with nontargeting short hairpin RNA (shRNA) or shRNA targeting mouse dUTPase. Transduced cells were infected with indicated virus, and reverse transcription-quantitative PCR (RT-qPCR) analysis was performed for mRNA transcripts of mouse dUTPase (left) and MHV68 ORF54 (right) 6 hpi. (C) RT-qPCR analysis of transcript levels of genes adjacent to ORF46 and ORF54 in WT MEFs 24 hpi. (D) UNGase assay demonstrates no enzymatic activity of 46.CM/54.CM-infected UNG^−/−^ MEFs lysate. (E) Mouse dUTPase knockdown fibroblast cells from panel B were infected with 46.CM/54.CM or MR MHV68 at an MOI of 10. Cell lysates were prepared 6 hpi and incubated with dUTP for 0 or 24 h at 37˚C. PCR was performed with the treated dUTP. The lack of amplification correlates with an enzymatically active dUTPase. (F) UNG^−/−^ mice or WT C57BL/6 mice were infected by the intranasal route with 1,000 PFU of the indicated viruses. Virus titer from lung homogenate was determined by plaque assay. Each symbol represents the titer per milliliter of lung homogenate in an individual mouse. The line indicates the geometric mean titer. The dashed line depicts the limit of detection at 50 PFU/ml of lung homogenate. Significance was determined by two-way unpaired *t* test on infected animals: *, *P* < 0.05; ****, *P* < 0.0001. Download FIG S3, EPS file, 1.0 MB. Copyright © 2018 Dong et al. 2018 Dong et al. This content is distributed under the terms of the Creative Commons Attribution 4.0 International license.

We next examined the impact of the combinatorial 46.CM/54.CM mutation on colonization of the spleen. Splenomegaly was significantly reduced in mice infected with 46.CM/54.CM compared to the MR virus 16 days after intranasal infection (Fig. 5D). There was a 2-log defect in the establishment of latency in the splenocytes of 46.CM/54.CM-infected mice compared to their MR-infected counterparts, confirmed by similar magnitudes of reduction in the frequency of reactivation from latency (Fig. 5E and F and Table 1 and Table 2). Upon intraperitoneal inoculation to bypass the restrictive tissue of the lung, we observed no defect in the establishment of latency or reactivation from latency in the spleen 16 dpi (Fig. 5G to I and Table 1 and Table 2) or PECs (Fig. S4A and B). Long-term latency maintenance was not impacted by the absence of vUNG and vDUT activities by either route (Fig. S4C to F). The route-dependent phenotype led us to examine the expression level of host dUTPase and host UNG. The spleen tissue had higher dUTPase transcript levels compared to the lung, suggesting that the host enzyme might compensate for the lack of the viral enzyme in the splenocytes (Fig. S5A and B). To summarize, in marked contrast with the single enzymatic vUNG and vDUT mutants, which reached 4.1× 10^5^ and 2.5× 10^4^ splenocytes, respectively, per mouse at 16 dpi, the combinatorial vUNG/vDUT enzymatic mutant was significantly impaired in the colonization of the spleen, reaching only 4.0× 10^3^ splenocytes per mouse; this is more than a100-fold reduction in the latent viral burden of the combinatorial mutant compared to WT virus in the animal (Table 2).

10.1128/mBio.01831-18.5 FIG S4 Loss of both vUNG and vDUT enzymatic activities does not impact viral latency establishment upon intraperitoneal infection or long-term latency maintenance. C57BL/6 mice were infected by either the intraperitoneal (IP [A to D]) or intranasal (IN [E and F]) route with 1,000 PFU of the indicated viruses. (A) Frequency of PECs harboring latent genomes at 16 dpi. (B) Frequency of PECs capable of reactivation from latency upon explant at 16 dpi. (C and E) Weights of spleens harvested at 42 dpi. (D and F) Frequency of splenocytes harboring latent genomes at 42 dpi. For the limiting dilution analyses, curve fit lines were determined by nonlinear regression analysis. Using Poisson analysis, the intersection of the nonlinear regression curves with the dashed line at 63.2% was used to determine the frequency of cells that were either positive for the viral genome or reactivating virus. For panels C and E, each symbol represents an individual mouse. For panels A, B, and D, the data were generated from at two independent experiments with 3 to 6 mice per group. For panel F, the data were generated from three independent experiments with 3 to 6 mice per group. Download FIG S4, EPS file, 0.3 MB. Copyright © 2018 Dong et al. 2018 Dong et al. This content is distributed under the terms of the Creative Commons Attribution 4.0 International license.

10.1128/mBio.01831-18.6 FIG S5 Higher transcript levels of murine UNG and murine dUTPase in splenocytes compared to lung cells. Lung cells and splenocyces were harvested from WT C57BL/6 mice, and the transcript levels of murine dUTPase (A) and UNG (B) were examined by RT-qPCR. Cells from each tissue were isolated from 3 or 4 mice. WT and UNG^−/−^ (KO) MEFs were used for internal controls. Significance was determined by two-way unpaired *t* test: ****, *P* < 0.0001. Download FIG S5, EPS file, 0.1 MB. Copyright © 2018 Dong et al. 2018 Dong et al. This content is distributed under the terms of the Creative Commons Attribution 4.0 International license.

### vUNG and vDUT promote viral genomic stability *in vivo*.

**FIG 6:**
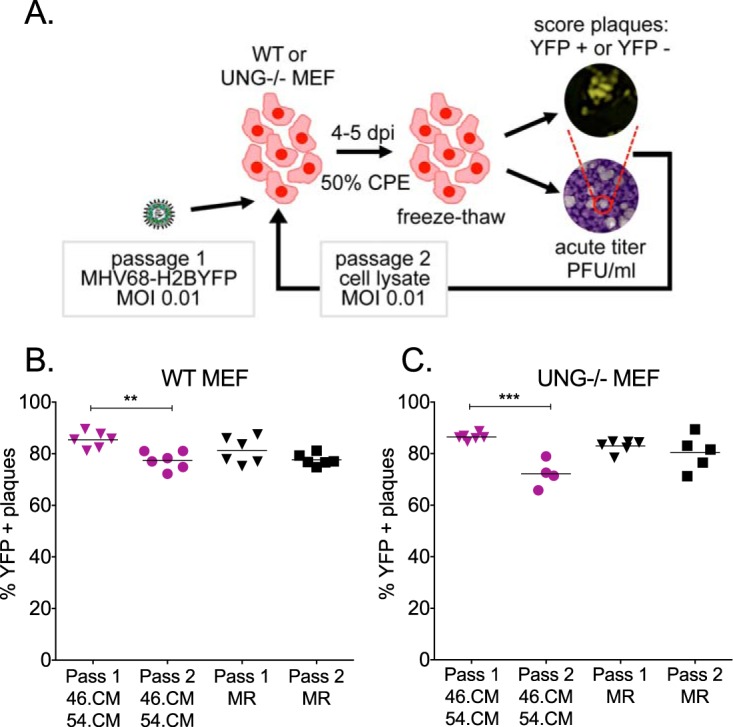
Loss of enzymatic activities of both MHV68 vUNG and vDUT leads to loss of YFP reporter gene in cell culture. (A) Schematic of serial passage experiment in MEFs. For passage 1, UNG^−/−^ or WT MEFs were infected at an MOI of 0.01 with the recombinant MHV68 lacking the enzymatic activity of both vUNG and vDUT (46.CM/54.CM) or the MR virus. Passage 1 viruses were harvested when 50% CPE was observed, and titers were quantified by plaque assay. Passage 1 viruses were then normalized to an MOI of 0.01 for the second passage of infection in the same cell type. (B and C) The percentage of YFP-positive plaques was quantified by fluorescence microscopy at 7 to 8 dpi. Samples were harvested following one or two passages in WT MEFs (B) or UNG^−/−^ MEFs (C). **, *P* < 0.01, and ***, *P* < 0.001, by two-way unpaired *t* test.

The synergistic defect we observed for the 46.CM/54.CM double mutant supports a model wherein both the vUNG and vDUT enzymatic activities must be lost to reveal the consequences of uracil incorporation in the viral genome. Thus, we reasoned that the replication defect might be attributable to loss of viral genomic integrity. Taking advantage of the CMVIEp-driven H2BYFP reporter gene that is incorporated in our recombinant viruses (37), we examined for loss of YFP fluorescence in the plaques produced upon serial passage of 46.CM/54.CM and MR viruses in MEFs (Fig. 6A). After two rounds of infection at a low multiplicity of infection (MOI) in WT MEFs, a significant 5% loss of YFP expression was observed in 46.CM/54.CM plaques between passage 1 and passage 2, but not in the MR infection (Fig. 6B). A greater loss of YFP in the plaques of 46.CM/54.CM compared to MR virus was also observed in UNG^−/−^ MEFs (Fig. 6C). Despite the loss of the YFP reporter gene, viral replication was not impacted by the second passage in either WT or UNG^−/−^ MEFs (data not shown).

**FIG 7:**
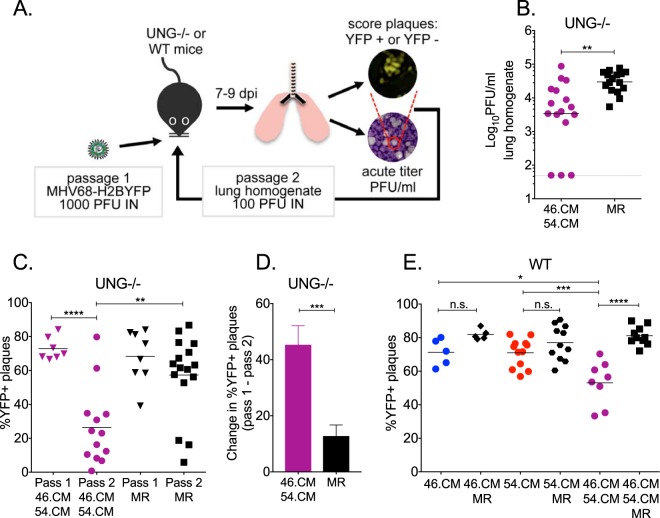
Loss of enzymatic activities of both MHV68 vUNG and vDUT leads to loss of YFP reporter gene *in vivo*. (A) Schematic of serial passage experiment in mouse lungs. For passage 1, UNG^−/−^ mice were infected by the intranasal route with 1,000 PFU with the recombinant MHV68 lacking the enzymatic activity of both vUNG and vDUT (46.CM/54.CM) or the MR virus. Titers of viruses recovered from passage 1 UNG^−/−^ animals 7 dpi were quantified by plaque assay. Passage 1 viruses were then normalized to 100 PFU for the second passage of infection in the UNG^−/−^ mice. WT mice were infected by the intranasal route with 1,000 PFU with the recombinant MHV68 lacking the enzymatic activity of vUNG (ORF46.CM), vDUT(ORF54.CM), or both vUNG and vDUT (46.CM/54.CM) and their MR viruses. Lung homogenates were prepared 9 dpi for plaque assay. (B) Passage 2 viruses in lung homogenates from UNG^−/−^ mice were determined by plaque assay at 9 dpi. The line indicates the geometric mean titer. Each symbol represents an individual mouse. The dashed line depicts the limit of detection at 50 PFU/ml of lung homogenate. (C) The percentage of YFP-positive plaques was quantified by fluorescence microscopy at 7 to 8 dpi for lung homogenate from infected UNG^−/−^ mice. Each symbol represents one mouse. (D) The increase in the percentage of YFP loss from passage 1 to passage 2. (E) WT C57BL/6 mice were infected by the intranasal route with 1,000 PFU of the indicated viruses. The percentage of YFP-positive plaques was quantified by fluorescence microscopy at 7 to 8 dpi. Each symbol represents one mouse. For panels B to D, significance was determined by two-way unpaired *t* test, and for panel E, significance was determined by one-way ANOVA: *, *P* < 0.05; **, *P* < 0.01; ***, *P* < 0.001; ****, *P* < 0.0001. n.s., not significant.

Next, we investigated the viral genomic stability in the context of acute replication in the lungs, wherein virus replication was restricted in the absence of MHV68 vUNG and vDUT enzymatic activities. UNG^−/−^ mice were infected via the intranasal route with 1,000 PFU for the first passage, followed by 100 PFU for the second serial passage due to reduced virus production in the 46.CM/54.CM lung homogenates from the first passage (Fig. 7A). Consistent with our previous findings in WT mice, replication of the 46.CM/54.CM virus was reduced by 1 log compared to the MR virus in lungs after the second passage (Fig. 7B). The plaques were also scored for the presence or absence of YFP expression by fluorescence microscopy. We observed a striking loss of YFP in the plaques derived from the lung homogenate of 46.CM/54.CM-infected mice. Approximately 70% of the plaques were YFP positive in virus recovered from one passage through the lungs, and this dropped to 30% in plaques from virus recovered from the second serial passage of 46.CM/54.CM (Fig. 7C and D). In contrast, there was only a slight, insignificant decrease in plaques expressing YFP in the homogenates recovered from lungs of MR-infected mice (Fig. 7C and D). We note that for the second passage, one to three mice were infected with lung homogenate derived from a single infected animal. A consistent rate of loss was observed for each independent lineage of the 46.CM/54.CM (∼40%) or the MR-passaged viruses (∼10%) (see Fig. S6A to C in the supplemental material). Interestingly, the instability observed for 46.CM/54.CM based on YFP loss was greater than that observed for either single mutant alone in WT mice, consistent with a synergistic role for vUNG and vDUT activities (Fig. 7E; Fig. S6D).

10.1128/mBio.01831-18.7 FIG S6 Infection with MHV68 mutant lacking both vUNG and vDUT enzymatic activities leads to loss of YFP reporter gene *in vivo*. (A to C) As described for Fig. 7, for passage 1, UNG^−/−^ mice were infected by the intranasal route with 1,000 PFU of the recombinant MHV68 lacking the enzymatic activity of both vUNG and vDUT (46.CM/54.CM) or the MR virus. Titers of viruses recovered from passage 1 animals 7 dpi were quantified by plaque assay. Passage 1 viruses were then normalized to 100 PFU for the second passage of infection in the UNG^−/−^ mice. (A and B) Data from Fig. 7C for 46.CM/54.CM (A) and MR virus (B) are graphed to illustrate the linkage of the YFP loss for recombinant viruses as they were passaged through UNG^−/−^ mice. The circles and squares represent the percentage of YFP-positive plaques in the inoculum used to infect the mice (input). Symbols (open, closed, or partial shading) in passage 1 represent the percentage of YFP-positive plaques in viruses isolated from the lungs of individual mice in the first round of infection. Symbols in passage 2 (open, closed, or partial shading) represent the percentage of YFP-positive plaques in viruses isolated from the lungs of individual mice that were infected with the inoculum of virus isolated from passage 1 mice. The virus from the first passage was used to infect multiple mice in the second passage. Matched symbol patterns linked by a line trace the lineage of these independent passages. (C) The mean of individual lineages in panels A and B. Matched symbol patterns linked by a line trace the lineage of these independent passages. (D) As described for Fig. 7, WT mice were infected by the intranasal route with 1,000 PFU of the recombinant MHV68 lacking the enzymatic activity of vUNG (ORF46.CM), vDUT(ORF54.CM), or both vUNG and vDUT (46.CM/54.CM) and their MR viruses. Lung homogenates were prepared 9 dpi for plaque assay. Data from Fig. 7E are graphed to illustrate the linkage of the YFP loss for recombinant viruses as they were passaged through WT mice as described for panel C. Download FIG S6, EPS file, 0.1 MB. Copyright © 2018 Dong et al. 2018 Dong et al. This content is distributed under the terms of the Creative Commons Attribution 4.0 International license.

**FIG 8:**
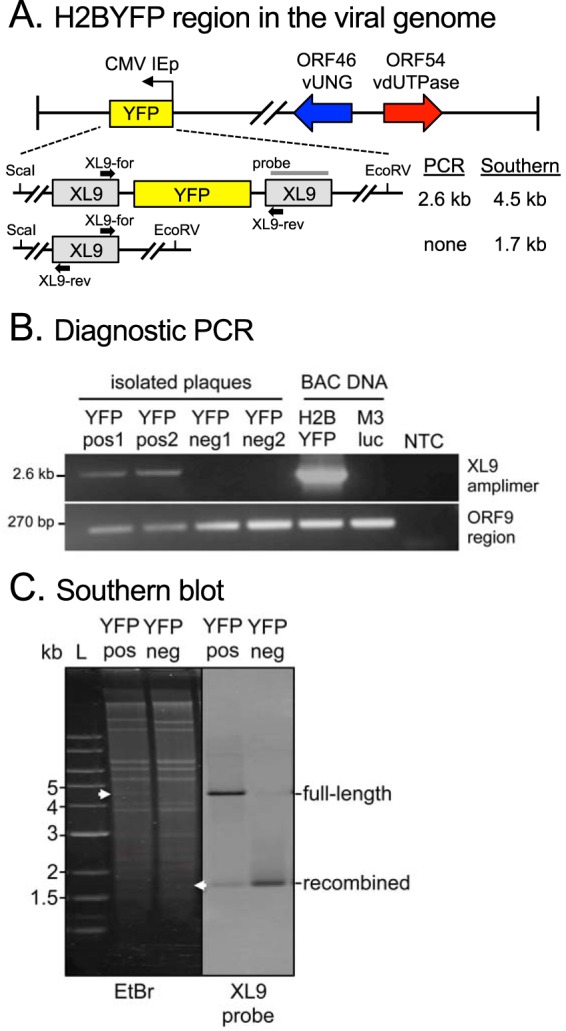
Loss of enzymatic activities of both MHV68 vUNG and vDUT results in higher YFP loss due to recombination. (A) Schematic of H2BYFP reporter gene position between two repeated XL9 elements in the MHV68 genome. The locations of primers for PCR and restriction enzyme sites and probe for Southern blotting are indicated; the expected sizes of PCR amplimers and restriction fragments are shown to the right. (B) PCR products from isolated YFP-positive and YFP-negative plaques are consistent with the presence or loss of the YFP reporter gene. ORF9 amplification serves as the template input control. Multiple YFP-positive and YFP-negative plaques (*n* > 20 for each) were screened with the representative gel shown here. (C) The Southern blot probe for XL9 demonstrates a shift in EcoRV/ScaI restriction fragment size in DNA isolated from YFP-positive and YFP-negative plaques, consistent with recombination between XL9 repeats and loss of the H2BYFP reporter gene.

The loss of YFP could be attributed to either single nucleotide polymorphisms (SNPs) in the YFP reporter gene or a more dramatic insertion or deletion in that genomic region. In MHV68-H2BYFP recombinant viruses, the H2BYFP gene is flanked by two XL9 insulators designed to prevent chromatin silencing of the CMVIEp-YFP reporter gene (37) (Fig. 8A). Herpesvirus genomes have been reported to undergo homologous recombination based on expansion and contraction of repeated elements upon passage in tissue culture (38, 39). We examined whether the YFP loss phenotype in the 46.CM/54.CM-infected mice involved recombination events between the XL9 repeats that flank the CMVIEp-H2BYFP reporter gene. We designed diagnostic primers that would only yield an amplimer if the YFP gene was present (Fig. 8A). The 2.6-kb CMVIEp-H2BYFP genomic region was only amplified from YFP-positive samples (Fig. 8B). Primers flanking the XL9 repeats amplified a 1.3-kb product in all YFP-negative samples that revealed a single intact XL9 repeat upon Sanger sequencing (data not shown). In addition, we performed whole-genome sequencing of 46.CM/54.CM and MR viruses isolated from the lung passage experiments in UNG^−/−^ mice (see Fig. S7 in the supplemental material). In total, ten 46.CM/54.CM and eight MR viruses were plaque purified and minimally expanded in fibroblasts prior to PCR-based enrichment of the entire MHV68 genome for next-generation sequencing (see Table S1 and Fig. S7A in the supplemental material). Viral genomes were assembled *de novo* via VirGA (40) (Fig. S7B) and aligned to their respective references using pairwise alignment with Mauve (41). The pairwise alignment confirmed YFP reporter gene loss in the YFP-negative plaques, but did not reveal any SNPs other than the intended mutations in ORF46 and ORF54 (data not shown).

10.1128/mBio.01831-18.8 FIG S7 Amplification and sequencing of the whole viral genome from plaques or infected lungs. (A) The schematic of amplification and sequencing of the whole viral genome from plaques or infected lung tissue. (B) The *de novo-*assembled genomes. Columns describe the total viral genome length and percentage of the viral genome with a coverage depth of 100 or more. Download FIG S7, EPS file, 0.3 MB. Copyright © 2018 Dong et al. 2018 Dong et al. This content is distributed under the terms of the Creative Commons Attribution 4.0 International license.

10.1128/mBio.01831-18.9 TABLE S1 Primers and gBlocks used in this study. Download Table S1, DOCX file, 0.1 MB. Copyright © 2018 Dong et al. 2018 Dong et al. This content is distributed under the terms of the Creative Commons Attribution 4.0 International license.

We also examined recombination by Southern blot hybridization of the ScaI/EcoRV-digested DNA isolated from a YFP-positive and YFP-negative plaque with an XL9 probe. A 4.5-kb fragment consistent with the full-length H2BYFP region flanked by two copies of XL9 was detected in the YFP-positive sample, while the recombined shorter 1.7-kb fragment was detected in the YFP-negative sample (Fig. 8C). The recombined fragment was also detected in the YFP-positive sample, indicating a low level of ongoing recombination at this locus. The loss of the YFP reporter gene in the serial passaging experiments is due to an accelerated rate of recombination in the absence of the vUNG and vDUT enzymatic activities. This is a striking indication that these two enzymes coordinate to maintain the integrity of the viral genome.

## DISCUSSION

Viral fitness and evolution are shaped by factors that influence genomic stability. Unrepaired uracils introduced by misincorporation or cytidine deamination that lead to mutations are problematic for DNA viruses or those with DNA intermediates (42, 43). DUT and UNG homologs are conserved across the herpesvirus family, and most exhibit their respective activity to metabolize uracil or excise it from DNA (6, 10, 16, 20, 21, 44, 45). To dissect the contributions from each of these two enzymes and explore potential synergistic functions, we mutated the enzymatic sites in MHV68 vUNG (ORF46) and vDUT (ORF54). Severe replication and pathogenesis defects were observed in the absence of both vUNG and vDUT activities, suggesting compensation when either is eliminated. The compromised replication and dissemination of the double enzymatic mutant were concordant with a loss of viral genomic stability, read out by an increase in recombination. These data indicate that vUNG and vDUT coordinate to stabilize the herpesvirus genome to promote pathogenesis *in vivo*.

Herpesviruses with a complete loss of vUNG protein are defective for replication in nonproliferating cells, including neurons (HSV-1), serum-starved human fibroblasts (HCMV), and lung tissue comprised of alveolar epithelial cells (MHV68) (10, 11, 15). These cells are presumed to have low host UNG activities and higher dUTP/dTTP ratios (6, 46), such that vUNG would be required to compensate for low host UNG to remove uracils. Thus, we expected that the ORF46.CM mutant would have a similar phenotype to the ORF46.stop mutant (10). However, in contrast to the elimination of vUNG expression in the ORF46.stop infection that led to severely compromised expansion in the lungs and dissemination, ablation of vUNG enzymatic activity (ORF46.CM) did not impact acute replication or latency in mice (Fig. 2 and 3). This agrees with previous reports for EBV vUNG and the poxvirus vaccinia virus vUNG, wherein vUNG expression is required for lytic replication but its enzymatic activity is dispensable (9, 47). Many herpesvirus vUNGs interact with viral DNA replication factors. HSV-1 vUNG and HCMV vUNG bind their respective viral DNA polymerases, in complex with the viral DNA polymerase processivity factor (17–19). EBV vUNG binds both the viral DNA polymerase BALF5 and the processivity factor BMRF1, in addition to the viral gene transactivator RTA (9). A mutation in EBV vUNG that reduces these interactions results in decreased reactivation upon transient transfection, suggesting that vUNG may promote lytic replication via an undefined scaffolding function. However, this mutant (H213L) is also defective for uracil excision (9). The ORF46.CM (D85N H207L) mutant characterized here partially overlaps with the EBV mutation, but we did not observe an impact on productive replication in cell culture, mouse lungs, or latent splenocytes upon explant. Further investigation with mutants that clearly dissect enzymatic activities from interactions with viral DNA replication and transcription factors will be required to understand the essential nonenzymatic role of vUNG that promotes replication.

In a previous report, silencing of host UNG2 or ectopic expression of UGI, a UNG inhibitor of the Bacillus subtilis bacteriophage that inhibits both viral and host UNG activities, led to a 2- to 3-fold reduction of EBV genome copy number upon reactivation in epithelial cells (16). KSHV genome copy numbers and virion production were reduced upon reactivation in primary effusion lymphoma cells upon silencing of host UNG2 (48). However, expression of UGI in the context of human melanoma MeWo cells infected with a VZV mutant lacking vUNG (ORF59) does not impair viral replication (12). To address whether the host UNG is compensating for the vUNG enzymatic activity of MHV68, we infected UNG^−/−^ mice. Productive replication of mutants lacking ORF46 or ORF46 enzymatic activity was indistinguishable between UNG^−/−^ or WT mice (Fig. 1; Fig. S1A). The lack of any additional phenotype in the UNG^−/−^ mice might be due to the low level of host UNG activity in the lungs, compensation by other UDGs, or the vUNG serving a nonoverlapping role distinct from its host counterparts.

Single ablation of MHV68 vDUT activity (ORF54.CM) transiently impaired acute replication 5 dpi, in turn reducing latency establishment in C57BL/6 mice (Fig. 4). In another study, the enzymatic activity of MHV68 vDUT was dispensable for acute replication in the lung and latency in the spleen of BALB/c mice (21). C57BL/6 mice are more restrictive than BALB/c mice for acute MHV68 replication in the lungs, but the molecular basis for these strain-dependent differences is not clear (49). The delayed kinetics of ORF54.CM replication might reflect a requirement for DUT activity to generate dTTP for DNA replication or be due to a high dUTP/dTTP ratio leading to increased uracil misincorporation that cannot be fully reversed by vUNG. Herpesviruses encode other proteins involved in nucleotide metabolism that are dispensable for replication in cell culture. The viral RNR large and small subunits produce dNDP, and TK phosphorylates thymidine in the salvage pathway, potentially bypassing the need for dUMP generated by DUT (43). A recombinant MHV68 lacking viral RNR or TK fails to establish latency upon upper respiratory infection, yet successfully colonizes the spleen after intraperitoneal inoculation (30–33). This parallels the route-dependent phenotype for ORF46.stop (10) and the ORF54.CM and 46.CM/54.CM mutants (Fig. 4 and 5). Taken together, our data along with previous findings indicate that herpesviruses require these viral factors to promote nucleotide metabolism in quiescent, primary cells.

We generated a recombinant virus lacking the enzymatic activities of both vUNG and vDUT (46.CM/54.CM). This double mutant was severely attenuated in acute replication in the lung and had a 100-fold decrease in establishment of splenic latency compared to MR upon intranasal infection (Fig. 5). To our knowledge, this is the first demonstration of a synergistic role for the enzymatic functions of the vUNG and vDUT in herpesvirus pathogenesis. We also observed that 46.CM/54.CM rapidly lost the nonessential YFP reporter gene in the viral genome upon passage in MEFs and serial passage in the lungs of mice such that 70% of the plaques had lost YFP compared to 40% of the MR virus by the second passage. In WT mice, we examined YFP loss from viruses produced in the lungs of mice infected with either single mutant compared to the double mutant in a single passage. The combinatorial 46.CM/54.CM mutant exhibited a striking 2-log replication defect at 9 dpi (Fig. 5), and a significantly larger portion (∼50%) of the plaques isolated from the lung homogenate had lost YFP expression compared to the MR virus (∼20%) (Fig. 7). In contrast, the single ORF46.CM and ORF54.CM mutants were not impaired for replication and had rates of YFP loss comparable to those of MR controls 9 dpi (Fig. 7). The deletion of YFP likely arose from recombination between the directly repeated XL9 CTCF-binding elements that flank the gene (Fig. 8). Therefore, vUNG and vDUT provide synergistic roles that are critical to maintain viral genomic stability and promote replication of herpesviruses in restrictive tissues.

The more frequent loss of YFP fluorescence in the 46.CM/54.CM mutant plaques in cell culture did not manifest as a replication defect. This is similar to an earlier report of an HSV-1 vUNG/vDUT mutant that had more frequent loss of a β-Gal reporter gene activity, with no defect in viral replication upon serial passage in fibroblasts (25). We propose that viruses with mutations or deletions in essential genes would have reduced fitness and would not be recovered in our plaque assays. Our use of a nonessential reporter gene flanked by sequences intended to prevent chromatin silencing was a fortuitous approach that enabled us to identify an aspect of instability, recombination at repetitive elements, not previously reported for these mutants.

Surprisingly, no SNPs were detected in whole-viral-genome sequencing. The lack of observed mutations may be attributed to the small sample size or the limited depth of coverage. Alternatively, U:A mismatches that arose upon uracil misincorporation might be resolved by DNA replication and leave no trace of mutations. This led us to directly examine uracil incorporation in the viral genome. First, we applied a PCR-based approach to examine the uracil level in the XL9 region that was recombined. In this assay, the relative uracil content is determined by the difference in PCR efficiency between the two polymerases: a high-fidelity *Pfu* polymerase that is not able to read through uracil in the template DNA and a mutant *Pfu* polymerase that can read through uracil in the DNA without hindrance (50). There was no consistent difference in the PCR efficiency of the XL9 genomic regions between virion DNA from 46.CM/54.CM and MR virus infections in culture. Additionally, we used an enzyme-linked immunosorbent assay (ELISA)-based method to measure abasic sites wherein uracils are removed from a DNA sample by purified UNG and then treated with an aldehyde-reactive probe (ARP). This ELISA was more sensitive in the detection of uracils in control DNA. However, a high background level was observed in the DNA isolated from virions, possibly due to ARP reactivity with multiple forms of aldehydic lesions in DNA. Taken together, we did not detect an increase in uracils, abasic sites, or SNPs in the viral genomes of the 46.CM/54.CM virus using the approaches described here. Detailed mechanistic studies will require a cell culture model that recapitulates our *in vivo* phenotype and the development of a quantitative assay to measure uracil content and other forms of DNA damage.

We note a higher incidence of YFP loss for the 46.CM/54.CM virus after a single passage in WT mice (50% YFP negative) compared to the first passage in UNG^−/−^ mice (30% YFP negative) (Fig. 7). We propose that the host UNG is one of the host repair factors that destabilizes the viral genome through its efforts to remove uracils. We speculate that vUNG might serve to protect the viral genome from host UNG-mediated recruitment and initiation of BER. In prokaryotes and some eukaryotes, a DUT deficiency results in increased mutation, recombination, and DNA fragmentation, processes which in most cases are suppressed by inhibiting UNG initiated base excision repair (51). These phenotypes are attributed to the induction of recombinational repair on DNA strand breaks initiated by excision of misincorporated uracils (52, 53). Consequences of host UNG-initiated BER that would be detrimental to the virus include homologous recombination at repetitive hot spots, a stall in replication fork progression, or the trigger of cellular checkpoints, as reported in prokaryotic and yeast systems (51, 54). In addition, the DNA mismatch repair factors are a backup strategy for uracil excision in B cells in the absence of UNG2 (55) and were recently reported to be recruited to HSV-1 replication forks (56). Alternate host UDGs, including TDG and SMUG1, could also recognize misincorporated uracils or uracils that arise from host cytidine deaminases (7).

The analysis of the single and combinatorial mutants led us to identify a synergistic loss of replication and increase in recombination that support a general model for the coordinated actions of the viral UNG and viral DUT during infection. When the virus infects nonproliferating cells in mucosal tissues with low host UNG and DUT levels, there is likely a high dUTP/dTTP ratio. In the context of a WT virus infection, vDUT lowers dUTP, while vUNG surveys the viral genome to rapidly excise uracils from the nascent DNA. Thus, when the enzymatic activities of vUNG and vDUT are lost, uracil levels would be expected to increase in the viral genome. The lack of evidence for overall elevated uracil in the viral genome of 46.CM/54.CM suggests that the introduction of a uracil is a rare event, yet leads to a detrimental consequence if not repaired by the vUNG. Misincoporated uracils at the replication fork during viral DNA replication may be recognized by host UDGs, leading to abasic sites and an increase in recombination events between repeated elements.

In summary, we utilized a murine gammaherpesvirus infection of mice as a model pathogenesis system to examine the roles of the enzymatic activities of vUNG and vDUT. Our *in vivo* analysis indicates a synergistic role provided by both vUNG and vDUT in promoting viral replication and latency establishment. Moreover, we took advantage of a nonessential YFP reporter gene and observed a striking increase in recombination between flanking repeated elements that led to its deletion. High-throughput sequencing of laboratory-derived strains and clinical isolates reveals that recombination occurs between repetitive elements in HSV-1, HCMV, and EBV (57–60). Short sequence repeats and longer repetitive elements within origins of lytic replication and terminal repeats are a hallmark of herpesvirus genomes (29, 57). Our data support a model wherein vUNG and vDUT coordinate to maintain viral genomic stability by reducing recombination-mediated deletions to promote viral pathogenesis.

## MATERIALS AND METHODS

### Mice and cells.

Wild-type C57BL/6 mice were purchased from Harlan/Envigo RMS (Indianapolis, IN) or Jackson Laboratories (Bar Harbor, ME) or were bred at the Stony Brook University Division of Laboratory Animal Research facility. UNG^−/−^ mice were obtained from the Jaenisch lab (MIT) (34) and crossed with wild-type C57BL/6 mice at the M. D. Anderson Cancer Center facility. Heterozygous UNG^+/−^ C57BL/6 mice were transferred to Stony Brook University and used to derive the UNG^−/−^ C57BL/6 mice and UNG^+/+^ C57BL/6 mice. All animal protocols were approved by the Institutional Animal Care and Use Committee of Stony Brook University. Murine embryonic fibroblasts (MEFs) were harvested and maintained in Dulbecco’s modified Eagle medium (DMEM) supplemented with 10% fetal bovine serum (FBS), 100 U/ml of penicillin, 100 mg/ml of streptomycin, and 2 μM l-glutamine (10% DMEM) at 37°C in 5% CO_2_. MEFs at passages 2 and 3 were used for viral growth curves and limiting dilution reactivation assays. Immortalized NIH 3T12 (ATCC CCL-164) and NIH 3T3 (ATCC CCL-163) cells were maintained in DMEM with 8% FBS, 100 U/ml of penicillin, 100 mg/ml of streptomycin, and 2 μM l-glutamine at 37°C in 5% CO_2_.

### Protein alignment.

UNG multiple pairwise alignments were generated using Geneious alignment (Geneious v10.1.2) with a BLOSUM90 cost matrix. dUTPase multiple pairwise alignments were built using ClustalW alignment with the BLOSUM cost matrix. For dUTPase motif III, alignments were manually adjusted based on the order of the identified enzymatic motif for gammaherpesvirus dUTPase (2). The GenBank accession numbers of UNG and dUTPase used in this study are listed in Table S2 in the supplemental material.

10.1128/mBio.01831-18.10 TABLE S2 GenBank accession numbers of UNG and dUTPase. Download Table S2, DOCX file, 0.1 MB. Copyright © 2018 Dong et al. 2018 Dong et al. This content is distributed under the terms of the Creative Commons Attribution 4.0 International license.

### Virus generation and sequencing analysis.

The recombinant MHV68-H2BYFP bacterial artificial chromosome (BAC) was kindly provided by Samuel Speck (37). The recombinant MHV68-M3Luc BAC was a gift from Ting-Ting Wu (35). MHV68 ORF46.stop virus and ORF46.stop marker rescue (MR) virus on the H2BYFP BAC were previously described (10) and then made on the M3Luc BAC in this study. Recombinant MHV68 BACs with mutations in ORF46 and ORF54 were generated by *en passant* mutagenesis as previously described (36). The primers and gene blocks (gBlocks) used to generate mutant viruses are listed in Table S1. For each of the mutants, the marker rescue (MR) viruses were made to repair the mutations back to wild-type. Virus passage and titer determination were performed as previously described (61, 62). To validate the sequences of our mutant viruses, BAC DNA was prepared using Qiagen columns (Germantown, MD), and a DNA library was prepared using Illumina Nextera DNA Library Preparation kit (Illumina, San Diego, CA). Fragmented and tagged DNA was subjected to Miseq at the Stony Brook Microarray Facility. Whole-genome sequencing analysis was performed as paired-end DNA-seq; reads were aligned against the reference using bowtie2 (v2.3.0) (63) with default parameters. Variant detection was monitored using the FreeBayes tool (v1.1.0) (64), such that variants should have a minimum depth coverage of 500 reads, where the mapping quality and base quality are at least 20 on the Phred quality scale. Sequencing results revealed three amino acid changes in M1(N25S), M2(S66P), and ORF21(A510V) in all viruses derived from M3Luc BAC.

### Virus growth curve.

MEFs were seeded at 1.2 × 10^5^ per well in 12-well plates 1 day prior to infection at either a high multiplicity of infection (MOI of 5) or low MOI (MOI of 0.05), in triplicate. Samples were stored at −80°C and subjected to three cryogenic disruptions prior to the determination of titer by plaque assay (10).

### Serial passage in cell culture.

UNG^+/+^ and UNG UNG^−/−^ MEFs were seeded at 2.4 × 10^5^ cells per well in 6-well plates 1 day prior to infection (MOI of 0.01) in six replicates. Infected MEFs were harvested when displaying 50% cytopathic effect (CPE) and cryogenically disrupted three times to release infectious virus particles. Samples were then subjected to the determination of titer by plaque assay with the following modification. Prior to fixation, YFP-positive and YFP-negative plaques were counted by fluorescence microscopy (Olympus CKX41 microscope with an X-cite series 170 fluorescence lamp; Olympus, Bridgeport, CT). The mean percentage of YFP-positive plaques was based on observations of 50 to 200 plaques for each of the six replicates. This process was repeated for the second passage.

### Virus infection and organ harvest.

Eight- to 12-week-old mice were inoculated by the intranasal route with 1,000 PFU recombinant MHV68 in 20 μl of 10% DMEM or by intraperitoneal injection in 0.5 ml 10% DMEM under isoflurane anesthesia. The inoculum dose was validated by plaque assay titration. For acute replication, lungs were isolated and homogenized using 1-mm zirconia/silica beads (BioSpec, Bartlesville, OK) in a Mini-BeadBeater (BioSpec) and diluted for titration by plaque assay. For latency and reactivation assays, spleens were homogenized, treated to remove red blood cells, and passaged through a 100-μm-pore nylon filter. For PECs, 10 ml of 10% DMEM was injected into the peritoneal cavity, and approximately 8 ml of medium was withdrawn and centrifuged, and then the pellet was resuspended in 10% DMEM.

### UNGase assay.

UNGase activity was determined as previously described (10) with the following modifications. Mouse lungs and spleens were disrupted by glass pestle and a 100-μm mesh, passed through a 100-μm filter, and collected by centrifugation at 600 × *g*. For cell culture lysates, mock- or MHV68-infected UNG^−/−^ cells were scraped 24 hpi, collected by centrifugation at 600 × *g*. Cell pellets were resuspended in HEPES-EDTA and subjected to sonication (Branson Sonifier 450; Branson, Danbury, CT). Lysates from the cells (5 μg) and mouse tissue (20 μg) were incubated with 1 pmol of 5′-Alexa 488-modified, 19-mer single-stranded DNA oligonucleotide containing a single uracil at 37°C for 15 min in reaction buffer. Reactions were stopped and separated by 7.4 M urea–15% (19:1) polyacrylamide gel and then imaged by a Typhoon 9500 scanner (GE Healthcare, Chicago, IL).

### *In vivo* passage experiment.

UNG^−/−^ or WT mice were inoculated via the intranasal route with the indicated MHV68-H2BYFP viruses at 1,000 PFU. On 7 to 9 dpi, the lungs were isolated and homogenized, and the virus titers were determined by plaque assay. YFP-positive and YFP-negative plaques were counted as described above. The mean percentage of YFP-positive plaques was based on observations from 100 to 400 plaques for each mouse. This process was repeated for UNG^−/−^ mice, except 100 PFU was used for the second passage inoculation and lungs were isolated 9 dpi due to the lower dose. The disrupted lung homogenate isolated from the first passage on one mouse was used as the inoculum of one to three mice for the second passage.

### *In vivo* imaging of mice.

Mice were inoculated via the intranasal route with the recombinant MHV68-M3Luc viruses. On 5 and 9 dpi, mice were anesthetized with isoflurane and intraperitoneally injected with 3 mg/mouse d-luciferin (Perkin Elmer, Waltham, MA). Mouse hair was removed by applying depilatory cream (Nair) to the chest area. Mice were then scanned via the IVIS Lumina II *in vivo* imaging system (Perkin Elmer) 5 min after injection. For quantitation, total flux radiance (photons per second) was measured in each region of interest using Living Imaging software (Perkin Elmer, v4.3.1).

### Limiting dilution PCR and limiting dilution assay.

Single-copy-sensitive nested PCR was used to determine the frequency of cells harboring the MHV68 viral genome as previously described (61, 62). Briefly, single-cell suspensions were prepared and six 3-fold dilutions of cells with 12 replicates per dilution were plated in the background of NIH 3T12 for overnight proteinase K digestion at 56°C. With 2 rounds of a total of 80 cycles of amplification targeting MHV68 gene ORF50, PCR products were run on agarose gel to score the PCR-positive wells. Standards with 0.1, 1, and 10 copies of ORF50-containing plasmids were included as controls. To determine the frequency of infected cells that spontaneously reactivate from latency upon explant, bulk splenocytes and PECs were prepared as single-cell suspensions. Cells were plated by twelve 2-fold dilutions, with 24 replicates per dilution, on monolayers of WT MEF cells. CPE was scored 2 to 3 weeks after plating (61, 62). Disrupted cells were also plated in parallel in each limiting dilution assay to monitor the preformed viral particles; it was determined in each independent assay that less than 1% of the CPE was contributed by preformed viruses.

### ORF46 antibody production.

To produce anti-ORF46 mouse polyclonal sera, recombinant histidine-tagged ORF46 was expressed in Escherichia coli, purified by Talon cobalt resin (Thermo Pierce) as per the manufacturer’s instructions, and dialyzed into phosphate-buffered saline (PBS). C57BL/6 mice were intraperitoneally immunized with 50 μg ORF46 in Imject alum adjuvant (Thermo Pierce). Mice were boosted with 25 μg ORF46 at 2-week intervals. After three immunizations, mice were euthanized and polyclonal serum was harvested. All animal manipulations were approved by the Institutional Animal Care and Use Committee of University of Texas M. D. Anderson Cancer Center.

### Antibodies and immunoblotting.

Total protein lysate was prepared in radioimmunoprecipitation assay (RIPA) lysis buffer (150 mM sodium chloride, 1.0% IGEPAL CA-630, 0.5% sodium deoxycholate, 0.1% sodium dodecyl sulfate, 50 mM Tris [pH 8.0]) supplemented with protease inhibitors and phenylmethylsulfonyl fluoride (PMSF). Protein concentration was quantified using a Bradford assay (Bio-Rad, Hercules, CA). Protein lysates were separated using SDS-PAGE and transferred to polyvinylidene fluoride membranes. ORF46 was detected using anti-ORF46 mouse sera described above, ORF59 was detected using affinity-purified chicken anti-peptide antibodies against MHV68 ORF59 (Gallus Immunotech, Fergus, Ontario, Canada) (65). Mouse sera harvested from MHV68-infected mice 28 dpi was used to detect lytic antigen. ORF65 antibody is a kind gift from Ren Sun (66). GAPDH (glyceraldehyde-3-phosphate dehydrogenase) antibody is from Sigma-Aldrich (St. Louis, MO). Detection was performed with horseradish peroxidase (HRP)-conjugated anti-mouse IgG (Sigma), anti-rabbit IgG (Sigma) or anti-chicken IgY (Gallus) with an enhanced chemiluminescence reagent (ECL; Thermo Scientific). Data were collected with the LAS 500 chemiluminescence imager (GE Healthcare).

### Diagnostic PCR for XL9 recombination.

DNA samples prepared from isolated plaque material from the passaging experiments were used for PCRs with primers specific for the XL9 element that flanks the CMV IEp-driven H2BYFP reporter gene (37) or ORF9 (Table S1) and Dynazyme *Taq* (Thermo) under the following cycling conditions: 94°C for 2 min, followed by 40 cycles of 94°C for 30 s, 60°C for 30 s, 72°C for 4 min, and 72°C for 10 min. PCR products were separated by 2% agarose gel electrophoresis in 1× Tris-acetic acid-EDTA buffer.

### Southern blot.

The XL9 probe was amplified using XL9-271 and XL9-986 primers (Table S1) with H2BYFP BAC DNA as the template and Dynazyme *Taq* (Thermo) under the following cycling conditions: 94°C for 2 min, followed by 30 cycles of 94°C for 30 s, 60°C for 30 s, 72°C for 1 min, and 72°C for 10 min. XL9 amplimers were purified by gel extraction and labeled via random priming with hexanucleotide, digoxigenin (DIG)-dUTP and Klenow enzyme at 37°C for 21 h.

Material from YFP-positive and YFP-negative plaques was used to infect NIH 3T12 cells. When 50% CPE was observed, genomic DNA was prepared (Qiagen) and digested with EcoRV and ScaI overnight at 37°C. Restriction digests were separated by 0.8% agarose gel electrophoresis in 0.5× Tris-acetic acid-EDTA at 30 V overnight. The gel was transferred overnight by capillary action to a positively charged nylon membrane (GE Healthcare) in 20× SSC (1× SSC is 0.15 M NaCl plus 0.015 M sodium citrate). The membrane was crossed-linked (UV Stratalinker 2400 auto-cross-link; Stratagene) and hybridized with the XL9 probe in Easy Hyb (Roche). The DIG-labeled probe was detected with alkaline phosphatase-conjugated antibody to DIG and the NBT/BCIP (nitroblue tetrazolium–5-bromo-4-chloro-3-indolylphosphate) substrate per the manufacturer’s instruction (DIG DNA labeling and detection kit; Roche, Indianapolis, IN).

### Statistical analyses.

All data were analyzed using GraphPad Prism (v.6). Statistical significance was analyzed using two-way unpaired *t* test or one-way ANOVA. For limiting dilution PCR and limiting dilution assay, frequency was determined from the nonlinear regression fit of the data where the regression line intersected 63.2% based on the Poisson distribution.

10.1128/mBio.01831-18.1 TEXT S1 Supplemental materials and methods. Download Text S1, DOCX file, 0.1 MB. Copyright © 2018 Dong et al. 2018 Dong et al. This content is distributed under the terms of the Creative Commons Attribution 4.0 International license.
