# Supplementary material for: Combinatorial Loss of the Enzymatic Activities of Viral Uracil-DNA Glycosylase and Viral dUTPase Impairs Murine Gammaherpesvirus Pathogenesis and Leads to Increased Recombination-Based Deletion in the Viral Genome
Source: mBio. 2018 Oct 30;9(5):e01831-18. doi: 10.1128/mBio.01831-18 (PMC6212821; doi:10.1128/mBio.01831-18)
Supplement: TABLE S1 [file mbo005184129st1.docx]

| **Table S1. Primers and gBlocks used in this study** | | |
| --- | --- | --- |
| **Mutant MHV68 generation** | | |
| **Mutant** | **Forward primer (5’ to 3’)** | **Reverse primer (5’ to 3’)** |
| ORF46.CM (H207L) | TCTGATCAATGGCAGTAAGCATCTTGTATTAAAGGCGCAACTCCCTTCCCCCCTGGCATCTCTTAGGGATAACAGGGTAATCGATTT | GTTTTGATGAGTAGCCACCAAGAGATGCCAGGGGGGAAGGGAGTTGCGCCTTTAATACAAGATGCCAGTGTTACAACCAATTAACC |
| **Mutant** | **gBlock** | |
| ORF46.CM  (D85N) | GTGATGCGCTGGGCATTTTCCTGCCCTCCTGAAAATGTTAGGGTTGTTATTCTGGGACAGAACCCCTATCATGGGGGCCAGGCTAGGGATAACAGGGTAATCGATTTATTCAACAAAGCCACGTTGTGTCTCAAAATCTCTGATGTTACATTGCACAAGATAAAAATATATCATCATGAACAATAAAACTGTCTGCTTACATAAACAGTAATACAAGGGGTGTTATGAGCCATATTCAACGGGAAACGTCTTGCTCGAGGCCGCGATTAAATTCCAACATGGATGCTGATTTATATGGGTATAAATGGGCTCGCGATAATGTCGGGCAATCAGGTGCGACAATCTATCGATTGTATGGGAAGCCCGATGCGCCAGAGTTGTTTCTGAAACATGGCAAAGGTAGCGTTGCCAATGATGTTACAGATGAGATGGTCAGACTAAACTGGCTGACGGAATTTATGCCTCTTCCGACCATCAAGCATTTTATCCGTACTCCTGATGATGCATGGTTACTCACCACTGCGATCCCCGGGAAAACAGCATTCCAGGTATTAGAAGAATATCCTGATTCAGGTGAAAATATTGTTGATGCGCTGGCAGTGTTCCTGCGCCGGTTGCATTCGATTCCTGTTTGTAATTGTCCTTTTAACAGCGATCGCGTATTTCGTCTCGCTCAGGCGCAATCACGAATGAATAACGGTTTGGTTGATGCGAGTGATTTTGATGACGAGCGTAATGGCTGGCCTGTTGAACAAGTCTGGAAAGAAATGCATAAGCTTTTGCCATTCTCACCGGATTCAGTCGTCACTCATGGTGATTTCTCACTTGATAACCTTATTTTTGACGAGGGGAAATTAATAGGTTGTATTGATGTTGGACGAGTCGGAATCGCAGACCGATACCAGGATCTTGCCATCCTATGGAACTGCCTCGGTGAGTTTTCTCCTTCATTACAGAAACGGCTTTTTCAAAAATATGGTATTGATAATCCTGATATGAATAAATTGCAGTTTCATTTGATGCTCGATGAGTTTTTCTAATCAGAATTGGTTAATTGGTTGTAACACTGGCGGGTTGTTATTCTGGGACAGAACCCCTATCATGGGGGCCAGGCCAATGGTTTGGCCTTTAGTGTTCATGAAAACTTTCCAGTC | |
| ORF46.CM MR  (D85N) | CTATCATGGGGGCCAGGCCAATGGTTTGGCCTTTAGTGTTCATGAAAACTTAGGGATAACAGGGTAATCGATTTATTCAACAAAGCCACGTTGTGTCTCAAAATCTCTGATGTTACATTGCACAAGATAAAAATATATCATCATGAACAATAAAACTGTCTGCTTACATAAACAGTAATACAAGGGGTGTTATGAGCCATATTCAACGGGAAACGTCTTGCTCGAGGCCGCGATTAAATTCCAACATGGATGCTGATTTATATGGGTATAAATGGGCTCGCGATAATGTCGGGCAATCAGGTGCGACAATCTATCGATTGTATGGGAAGCCCGATGCGCCAGAGTTGTTTCTGAAACATGGCAAAGGTAGCGTTGCCAATGATGTTACAGATGAGATGGTCAGACTAAACTGGCTGACGGAATTTATGCCTCTTCCGACCATCAAGCATTTTATCCGTACTCCTGATGATGCATGGTTACTCACCACTGCGATCCCCGGGAAAACAGCATTCCAGGTATTAGAAGAATATCCTGATTCAGGTGAAAATATTGTTGATGCGCTGGCAGTGTTCCTGCGCCGGTTGCATTCGATTCCTGTTTGTAATTGTCCTTTTAACAGCGATCGCGTATTTCGTCTCGCTCAGGCGCAATCACGAATGAATAACGGTTTGGTTGATGCGAGTGATTTTGATGACGAGCGTAATGGCTGGCCTGTTGAACAAGTCTGGAAAGAAATGCATAAGCTTTTGCCATTCTCACCGGATTCAGTCGTCACTCATGGTGATTTCTCACTTGATAACCTTATTTTTGACGAGGGGAAATTAATAGGTTGTATTGATGTTGGACGAGTCGGAATCGCAGACCGATACCAGGATCTTGCCATCCTATGGAACTGCCTCGGTGAGTTTTCTCCTTCATTACAGAAACGGCTTTTTCAAAAATATGGTATTGATAATCCTGATATGAATAAATTGCAGTTTCATTTGATGCTCGATGAGTTTTTCTAATCAGAATTGGTTAATTGGTTGTAACACTGGC | |
|  | **gBlock 1** | **gBlock 2** |
| ORF46.CM MR  (H207L) | CTGATCAATGGCAGTAAGCATCTTGTATTAAAGGCGCAACATCCTTCCCCCCTGGCATCTCTTAGGGATAACAGGGTAATCGATTTATTCAACAAAGCCACGTTGTGTCTCAAAATCTCTGATGTTACATTGCACAAGATAAAAATATATCATCATGAACAATAAAACTGTCTGCTTACATAAACAGTAATACAAGGGGTGTTATGAGCCATATTCAACGGGAAACGTCTTGCTCGAGGCCGCGATTAAATTCCAACATGGATGCTGATTTATATGGGTATAAATGGGCTCGCGATAATGTCGGGCAATCAGGTGCGACAATCTATCGATTGTATGGGAAGCCCGATGCGCCAGAGTTGTTTCTGAAACATGGCAAAGGTAGCGTTGCCAATGATGTTACAGATGAGATGGTCAGACTAAACTGGCTGACGGAATTTATGCCTCTTCCGACCATCAAGCATTTTATCCGTACTCCTGATGATGCATGGTTACTCACCACTG | CTCCTGATGATGCATGGTTACTCACCACTGCGATCCCCGGGAAAACAGCATTCCAGGTATTAGAAGAATATCCTGATTCAGGTGAAAATATTGTTGATGCGCTGGCAGTGTTCCTGCGCCGGTTGCATTCGATTCCTGTTTGTAATTGTCCTTTTAACAGCGATCGCGTATTTCGTCTCGCTCAGGCGCAATCACGAATGAATAACGGTTTGGTTGATGCGAGTGATTTTGATGACGAGCGTAATGGCTGGCCTGTTGAACAAGTCTGGAAAGAAATGCATAAGCTTTTGCCATTCTCACCGGATTCAGTCGTCACTCATGGTGATTTCTCACTTGATAACCTTATTTTTGACGAGGGGAAATTAATAGGTTGTATTGATGTTGGACGAGTCGGAATCGCAGACCGATACCAGGATCTTGCCATCCTATGGAACTGCCTCGGTGAGTTTTCTCCTTCATTACAGAAACGGCTTTTTCAAAAATATGGTATTGATAATCCTGATATGAATAAATTGCAGTTTCATTTGATGCTCGATGAGTTTTTCTAATCAGAATTGGTTAATTGGTTGTAACACTGGCATCTTGTATTAAAGGCGCAACATCCTTCCCCCCTGGCATCTCTTGGTGGCTACTC~~A~~TCAAAAC |
| ORF54.CM  (H80A, D85N) | GGTCTGTTTATCCAGAAAACAAGCGGATTTCCCTTGATCCTAAATGCGATTTGTGCCAACAGTCTCGTCACCGGCGCAACTGGACTGATTAATCCCGGCTACCGGGGTGAAATTAGGGATAACAGGGTAATCGATTTATTCAACAAAGCCACGTTGTGTCTCAAAATCTCTGATGTTACATTGCACAAGATAAAAATATATCATCATGAACAATAAAACTGTCTGCTTACATAAACAGTAATACAAGGGGTGTTATGAGCCATATTCAACGGGAAACGTCTTGCTCGAGGCCGCGATTAAATTCCAACATGGATGCTGATTTATATGGGTATAAATGGGCTCGCGATAATGTCGGGCAATCAGGTGCGACAATCTATCGATTGTATGGGAAGCCCGATGCGCCAGAGTTGTTTCTGAAACATGGCAAAGGTAGCGTTGCCAATGATGTTACAGATGAGATGGTCAGACTAAACTGGCTGACGGAATTTATGCCTCTTCCGACCATCAAGCATTTTATCCGTACTCCTGATGATGCATGGTTACTCACCACTG | CTCCTGATGATGCATGGTTACTCACCACTGCGATCCCCGGGAAAACAGCATTCCAGGTATTAGAAGAATATCCTGATTCAGGTGAAAATATTGTTGATGCGCTGGCAGTGTTCCTGCGCCGGTTGCATTCGATTCCTGTTTGTAATTGTCCTTTTAACAGCGATCGCGTATTTCGTCTCGCTCAGGCGCAATCACGAATGAATAACGGTTTGGTTGATGCGAGTGATTTTGATGACGAGCGTAATGGCTGGCCTGTTGAACAAGTCTGGAAAGAAATGCATAAGCTTTTGCCATTCTCACCGGATTCAGTCGTCACTCATGGTGATTTCTCACTTGATAACCTTATTTTTGACGAGGGGAAATTAATAGGTTGTATTGATGTTGGACGAGTCGGAATCGCAGACCGATACCAGGATCTTGCCATCCTATGGAACTGCCTCGGTGAGTTTTCTCCTTCATTACAGAAACGGCTTTTTCAAAAATATGGTATTGATAATCCTGATATGAATAAATTGCAGTTTCATTTGATGCTCGATGAGTTTTTCTAATCAGAATTGGTTAATTGGTTGTAACACTGGCAACAGTCTCGTCACCGGCGCAACTGGACTGATTAATCCCGGCTACCGGGGTGAAATCTCTGTTATCCTGGCAACGGCGGCTCAGAGCACGGTGGAGATTCAACCGGGCCAATTG |
| ORF54.CM MR  (A80H, N85D) | GGTCTGTTTATCCAGAAAACAAGCGGATTTCCCTTGATCCTAAATGCGATTTGTGCCAACAGTCTCGTCACCGGCCACACTGGACTGATTGACCCCGGCTACCGGGGTGAAATTAGGGATAACAGGGTAATCGATTTATTCAACAAAGCCACGTTGTGTCTCAAAATCTCTGATGTTACATTGCACAAGATAAAAATATATCATCATGAACAATAAAACTGTCTGCTTACATAAACAGTAATACAAGGGGTGTTATGAGCCATATTCAACGGGAAACGTCTTGCTCGAGGCCGCGATTAAATTCCAACATGGATGCTGATTTATATGGGTATAAATGGGCTCGCGATAATGTCGGGCAATCAGGTGCGACAATCTATCGATTGTATGGGAAGCCCGATGCGCCAGAGTTGTTTCTGAAACATGGCAAAGGTAGCGTTGCCAATGATGTTACAGATGAGATGGTCAGACTAAACTGGCTGACGGAATTTATGCCTCTTCCGACCATCAAGCATTTTATCCGTACTCCTGATGATGCATGGTTACTCACCACTG | CTCCTGATGATGCATGGTTACTCACCACTGCGATCCCCGGGAAAACAGCATTCCAGGTATTAGAAGAATATCCTGATTCAGGTGAAAATATTGTTGATGCGCTGGCAGTGTTCCTGCGCCGGTTGCATTCGATTCCTGTTTGTAATTGTCCTTTTAACAGCGATCGCGTATTTCGTCTCGCTCAGGCGCAATCACGAATGAATAACGGTTTGGTTGATGCGAGTGATTTTGATGACGAGCGTAATGGCTGGCCTGTTGAACAAGTCTGGAAAGAAATGCATAAGCTTTTGCCATTCTCACCGGATTCAGTCGTCACTCATGGTGATTTCTCACTTGATAACCTTATTTTTGACGAGGGGAAATTAATAGGTTGTATTGATGTTGGACGAGTCGGAATCGCAGACCGATACCAGGATCTTGCCATCCTATGGAACTGCCTCGGTGAGTTTTCTCCTTCATTACAGAAACGGCTTTTTCAAAAATATGGTATTGATAATCCTGATATGAATAAATTGCAGTTTCATTTGATGCTCGATGAGTTTTTCTAATCAGAATTGGTTAATTGGTTGTAACACTGGCAACAGTCTCGTCACCGGCCACACTGGACTGATTGACCCCGGCTACCGGGGTGAAATCTCTGTTATCCTGGCAACGGCGGCTCAGAGCACGGTGGAGATTCAACCGGGCCAATTG |
| 46.CM/54.CM | Use the combination of ORF46.CM (D85N), ORF46.CM (H207L) and ORF54.CM (H80A, D85N) | |
| 46.CM/54.CM MR | Use the combination of ORF46.CM MR (N85D), ORF46.CM MR (L207H) and ORF54.CM MR (A80H, N85D) | |
| **XL9 identification primers** | | |
|  | **Forward primer (5’ to 3’)** | **Reverse primer (5’ to 3’)** |
| **Diagnostic** | **XL9-for:** GTTGTGTATCACTACCCATTGGC | **XL9-rev:** CTCAGTCCCTTAAGAGCTCTGTC |
|  | **ORF9-for:** CAATTGCTGTATCCCATCTGCG | **ORF9-rev:** GGAAACCCACATTCACCCAAAC |
| **Probe** | **XL9-986:** TCTCAACACCTGAAAGAGACTGG | **XL9-271:** TTCCTTTCAGTGTCCAAGTGTCA |
| XL9 amplimer | TCTCAACACCTGAAAGAGACTGGGCTTGAACAGTGAGGACTACATTAACCAGGGGGCTGGATCTGCATTGCTCACACTTGAGCAGAGATTGGAATCCAGCTGACTGTGAATGAGTGTGGAAGAGCCCCCTGCTGGAGGCAAATGACTGGAGCCCTGTGCATATGCTGGGCCATGAGGAAGAGCACAAGTATATTCTCTGGGACAGAGCTCTTAAGGGACTGAGCTAAGTCAGCCAGCATCCTCCTCCTCCTCACCCCACCCAATGGTAAATCTGATAAATCAGTGCTAATTTGCTTTGTATGTTAGAATTTACTAAATGTGATGGCTGATATTTTGAGCCAGGGTGGCAAATAAAGCTACTGACTCAAGAGCAATGTTTAGTGGTAACTTGGGTATGAGAATAGGATTGTGAATTGGTATGCAACTTTACCAGTGACAGTTGTGTATCACTACCCATTGGCATATATGTTCTAGTCTAGTGCTGAGAAAGATACACAAAATATTAAGACTTTTACAGCAAGATTGGTACCACAAAATGCTCATTTTTAGCTCTCATGGATTTTCTGTCTTGTAGTTTGTTGTAGCTTTCTCACTCTTTTTGAGGCATCACCAAGTCAACTTGTAAATTCTGTAATCACTTTCTTTCAGTAGATTTCTTATATCTAAATTTTAATTAAGACTAGGAATCCTTTTTGACACTTGGACACTGAAAGGAA | |
| **qPCR primers** | | |
| **Gene** | **Forward primer (5’ to 3’)** | **Reverse primer (5’ to 3’)** |
| ORF45 | CTCAGTATTGCAGGGAGGTCATC | TGACGATACCCAGCAGACTTTC |
| ORF47 | GTCTCTAAACCTGCTGACCACG | ACTTCCTGTCCTCCATAACTGAG |
| ORF53 | TGCGTAGATCAAAAAGACACCAC | TTTGATGTGTGCCTTTGTCTGTG |
| ORF55 | AGCAATGTCAGTGGCCCATG | TGATATCATTGGCTGCTGGGG |
| ORF54 | CCATCTATATCCTGCCTGTGAGC | GAGCAATGCCAGATAAATGGGTC |
| mDUT | TATCACCCATGGAGAAAGCCATC | TTCCCAAAGTTAAACAGCACGAC |
| β-actin | GGCACCACACCTTCTACAATG | GGGGTGTTGAAGGTCTCAAAC |
| **dUTPase assay PCR primers** | | |
| **Gene** | **Forward primer (5’ to 3’)** | **Reverse primer (5’ to 3’)** |
| ORF54 | TGGAATACTCCTTTGTGCCCAAG | CTTCGTGCGGAACCCTAATAAAC |
| **Multiplex primers for Mi-seq** | | |
| **Primer Name** | **Forward primer (5’ to 3’)** | **Reverse primer (5’ to 3’)** |
| BAC_2 | ATCAGACCGACGATACGAGTGG | TACCTGGAGTTTTTCCCACGGT |
| BAC_3 | AACTGGCGAGGAAGCAAAGAAG | GGCCGTAATATCCAGCTGAACG |
| BAC_5 | TGTGTCTTTTTACAGCGTCGGG | TTCCATGGGTGCATATTTGGGC |
| BAC_6 | GCAGCCACATCAAGCATACCAA | TGATGGGGTGTGGACTTCAACT |
| BAC_7 | GGACCTGCAGCACATTTTGAGA | GACCGCCAGGAAAAAGAAACCA |
| BAC_8 | CGGCCTCACATCATCATTTGCT | GACAAGCTTGCTGCCAAACATG |
| BAC_9 | TGCAGACTCTGAAGTGCTGACT | TTGCCAGATGTAATAGGCCCCA |
| BAC_10 | CGGTCCCTACATGCACTTCCTA | AGGCTTTGGATGTGCTCCCTAT |
| BAC_11 | AGATTGGACAGTGGAGGAAGCA | GAGCTGACCACATTCACCACAG |
| BAC_12 | GGACCTGTCAGACATGATGGCT | GGCCAGCTGCTGTTTATCCAAT |
| BAC_13 | TGGCTGGCCAAAAGAAAAGAGA | GCTCATCGGGCTTAAAGTCCAC |
| BAC_14 | TATCTGCACTAGACCAGGCCTG | CAATATGGTGTGCCCCTCCCTA |
| BAC_16 | AAGAGGGTAAGCTGGTGGACAA | CAGGGCGGCCTTTACAGTAATG |
| BAC_17 | AGCTTGAACTTGCCAACATCCC | GCCGGGAAATCTGTGCTCATTT |
| BAC_18 | GCATCGGTCAGTTGTTAGGAGG | CTGTGAGAAAGCCAGACACACC |
| BAC_19 | TGAGGTGGAGCAGACTAGTCAG | AATTGAAAGGCCGCCTACCATG |
| BAC_20 | AATTGTGACCTTGAAACTATTGATGCT | CTCCTCTCCAGGCAAGTTCTGA |
| BAC_21 | GCTCTAAAGATGCTATGCCATCCA | CAGGAGACCCTTGTGACTAGCA |
| BAC_22 | GGCACTGATGTTTTCTTGGAGC | GAGGAGGATGCTGGCTGACTTA |
| BAC_24 | AATGCATGAGCTCGGTACCAAG | GCGCACATTGGAAAGGTACACT |
| BAC_25 | CCTCGCTGGAGACTCCCTAAAT | GCTACGTAGCCCACCAAAAACA |
| BAC_26 | GAACCAGGCCAGCAGAATGTTT | GTCCCCTGGCGTGAATTTAGTG |
| BAC_27 | AAGCCATCGTTTATTGCACCCC | GCTCTAAATGCCCCTCACCAAC |
| BAC_28 | ACTTTGAACTAATTTCTGAAAACACTGGA | TATCATTCACCAGGTCGGGTCC |
| BAC_29 | GGGAATAAACATAACTTCACCTGGGT | AGGCCCTGGTGACAAAGTCATA |
| BAC_30 | ACAGTGAACTTAATGTTTTGAGGATCACT | CATGCCCTGAGCCTTGACAATC |
| BAC_31 | GATGGCTCATCAATGGTGCTGG | CTCCAGCTGGACACTTCGAGTA |
| BAC_32 | TGTACACAAACACTCATGGTATGTTCT | ACACAGTCTGGTGGGATGTTGA |
| BAC_33 | GGCATATCCAGAGAAGTTGAGGC | TCGCCTCTGGTGTCATAGATGG |
| BAC_34 | TATCCCAGCTGACCCTTCAACC | CCAAGGATAAGCAGGTCCTGGA |
| BAC_35 | ACCCAAGGTAAATCTCTTGATGTGC | TCGGAGAGTCCTCTACACTCGA |
| BAC_36 | GCTTTGCCAATAAGTGCAACAACA | GAGCGACCTTAGATCAGGGTGT |
| BAC_37 | TGGTGGGTTCCCTGTCTTTCTT | AATCTTGCGACCCAACATGACC |
| BAC_38 | CCCAGTTACACTCCCTAAGGGT | TGTCAGCGATCAGGAAGTCTGT |
| BAC_39 | AGGTGTCAATAATCTGTCAATCTCTGG | ATGCCGCCTTTAAGATTGTGGG |
| BAC_40 | TCTCGAAAGGATGTTCAAGTTGCT | TGAGGTCGTTTAAGTGGCTGGA |
| BAC_41 | TTATTGATGAGACATGGCCCGAG | TGTAATGGATTGGTGGCATGGC |
| BAC_42 | AAACACAACCGTTGATCCCACG | CATCATCCTCGTGTTTTGGGGG |
| BAC_43 | ACCTCTAGGGACAAGGCACAAT | GCCTGCGATGTGAATTTGGAGA |
| BAC_44 | AACCTCAGGAAATCCTCGTCCT | AGAGCTCGTTCTTGCTAAACGC |
| BAC_45 | CAGTGTGCCTATGACTAATAAACCAGG | AAAATTTATTGCTGAGAAAGACGAGATACA |
| BAC_47 | TGGGAGTGGTTTATGAGAGGGT | GAGCAGCCATCCACAAGTACAC |
| BAC_48 | TCTGAGACCCTTGTCCCTGTTG | TGGCTTTGGAGAGCTAGGAACA |
| BAC_49 | ATTGTGGGTTGGATTGGTCGAG | TGGAGGGTTTTCCAGACCGATT |
| BAC_50 | TGAGGGACAGTACCAGTAACACTG | AATCCTCACGTGCCAATTCACC |
| BAC_51 | GGTATTAGTTAGCAACTTGAATAGGGCA | ACGTCTGGGCAATCCAAGTCTA |
| BAC_52 | TGACAGTATTAGTCTGTTAAGGACATGT | CGGCAGGTATTCTGGTCTCTGA |
| BAC_55 | CTCACTATCCCACCTCCATGATG | CTCAGTCCCTTAAGAGCTCTGTC |
| BAC_56 | GTTGTGTATCACTACCCATTGGC | AACCGTGGTATCTAACACTGTCC |
| BAC_57 | CTCACTATCCCACCTCCATGATG | AACCGTGGTATCTAACACTGTCC |
