# Supplementary material for: Combinatorial Loss of the Enzymatic Activities of Viral Uracil-DNA Glycosylase and Viral dUTPase Impairs Murine Gammaherpesvirus Pathogenesis and Leads to Increased Recombination-Based Deletion in the Viral Genome
Source: mBio. 2018 Oct 30;9(5):e01831-18. doi: 10.1128/mBio.01831-18 (PMC6212821; doi:10.1128/mBio.01831-18)
Supplement: TABLE S2 [file mbo005184129st2.docx]

| **Table S2. GenBank accession numbers of UNG and dUTPase** | | | | | |
| --- | --- | --- | --- | --- | --- |
| **UNG** | | | **dUTPase** | | |
| **GenBank Name** | **Reference Name** | **Accession Number** | **GenBank Name** | **Reference Name** | **Accession Number** |
| uracil DNA glycosylase [Murid gammaherpesvirus 4] | MHV68 ORF46 | CAA70280.1 | deoxyuridine triphosphatase [Murid gammaherpesvirus 4] | MHV68 ORF54 | CAA70269.1 |
| ORF46 [Human gammaherpesvirus 8] | KSHV ORF46 | ALH45513.1 | ORF54 [Human gammaherpesvirus 8] | KSHV ORF54 | ALH45524.1 |
| uracil-DNA glycosylase [Human herpesvirus 4] | EBV BKRF3 | YP_401679.1 | deoxyuridine triphosphatase [Human gammaherpesvirus 4] | EBV BLLF3 | AHA36438.1 |
| uracil-DNA glycosylase [Human betaherpesvirus 5] | HCMV UL114 | APG57296.1 | N.A. | N.A. | N.A. |
| uracil-DNA glycosylase [Human alphaherpesvirus 1] | HSV-1 UL2 | YP_009137076.1 | deoxyuridine triphosphatase [Human alphaherpesvirus 1] | HSV-1 UL50 | AOY34293.1 |
| uracil-DNA glycosylase isoform b [Mus musculus] | Mouse UNG | NP_035807.2 | deoxyuridine triphosphatase [Mus musculus] | Mouse dUTPase | AAH53693.1 |
| uracil-DNA glycosylase isoform UNG2 [Homo sapiens] | Human UNG | NP_550433.1 | deoxyuridine triphosphatase [Homo sapiens] | Human dUTPase | AAC51123.1 |
