# Supplementary material for: Combinatorial Loss of the Enzymatic Activities of Viral Uracil-DNA Glycosylase and Viral dUTPase Impairs Murine Gammaherpesvirus Pathogenesis and Leads to Increased Recombination-Based Deletion in the Viral Genome
Source: mBio. 2018 Oct 30;9(5):e01831-18. doi: 10.1128/mBio.01831-18 (PMC6212821; doi:10.1128/mBio.01831-18)
Supplement: TEXT S1 [file mbo005184129s1.docx]

**Text. S1 Supplemental Material and Methods**

**Knockdown murine dUTPase with shRNA.** HEK 293T (ATCC#CRL-3249) cells were maintained in 10% DMEM at 37°C in 5% CO_2_. Non-targeting shRNA or shRNA targeting murine dUTPase (TRCN0000288187) were purchased from Sigma-Aldrich. Recombinant lentiviruses were produced by transient transfection in 293T cells using MISSION Lentiviral Packaging Mix (Sigma) and viral particles were harvested 48 hours post transfection and filtered through 0.45 μm filters. Transduced NIH 3T3 cells were selected in the presence of 1.5 μg puromycin for two weeks before using for experiments.

**DUTPase assay.** DUTPase activity in the infected cell lysates were based on the protocol previously described (1) with minor modifications. Briefly, shRNA transduced NIH 3T3 cells were infected with MHV68 at an MOI of 10. Cell lysates were harvested 6 hpi and disrupted in lysis buffer (100 mM Tris-HCl [pH 7.5], 50 mM NaCl, and 1.0% IGEPAL CA-630) supplemented with protease inhibitors (Roche) and PMSF for 15 mins on ice, then clarified at 4°C for 15 minutes at 8000 X g). Protein was quantified by Bradford assay (Bio-rad) and 3 ug was incubated with 5 mM dUTP (Thermo) at 37°C in Reaction Buffer (50 mM Tris [pH 7.5], 10 mM MgCl2, 10 mM DTT, and 0.1 mg/mL BSA). Reactions were stopped by rapid shift to -20°C and inactivation at 95°C for 10 min. Non-digested dUTP is at a final concentration of 1.25 mM. PCR was conducted using primers targeting a segment in ORF54 **(Table S1)** from MHV68, with the potentially digested dUTP from the dUTPase assay reaction, dGTP, dATP, and dCTP (200 mM final concentration each). Cycle conditions were 94°C 2 min; 95°C 30 s, 58°C 30 s, 72°C 1 min for 20 cycles; 72°C 10 minutes as final extension. PCR products were separated on a 1% agarose gel.

**Quantitative RT-PCR.** Quantitative RT-PCR was conducted as previously described (2) with the primers targeting ORF45, ORF47, ORF53, ORF55, ORF54, murine dUTPase and β-actin listed in Table S1. Primers targeting murine UNG and GAPDH were as previously described (2). The cycle threshold (CT) determination was within the linear range of detection for each amplimer. Relative quantitation was calculated based on the ∆∆CT method normalized by GAPDH or β-actin. Murine dUTPase and murine UNG in mouse tissues were measured by absolute quantification, as the copy numbers were calculated based on a standard curve generated using control plasmids bearing each target gene.

**Viral Genome amplification and NGS.** Genomic DNA from YFP^+^ or YFP^-^ infected NIH 3T12 cells was prepared using DNeasy Tissue and Blood kit (Qiagen). DNA was quantitated using Nanodrop (Thermo Scientific 2000c) and 100 ng of genomic DNA or 10 ng of BAC DNA was used as template in multiplex PCR reaction with Phusion U Hot Start PCR master mix (Thermo Scientific). Multiplex primers were designed using Primal Scheme with 2,500 bp per amplimer with 50 bp overlaps (3) **(Table S1)**. Ten pairs of primers were pooled together per PCR reaction and two independent pooling strategies were used per DNA to prevent bias of primers usage. Cycle conditions were 98°C 30 s; 98°C 10 s, 67°C 30 s, 72°C 40 s for 25 cycles with final extension at 72°C for 10 min. PCR products were then purified using QIAquick PCR purification kit (Qiagen) and ten PCR reactions per genomic DNA were pooled together as one sample. DNA library was prepared using Illumina Nextera DNA Library Preparation kit, then the DNA was fragmented and indexed for 2 X 150 cycles paired-end Miseq (Illumina, San Diego, CA).

**Sequence assembly with SNP and translocation analysis.** Sequences were assembled using the VirGA-Pipeline designed previously (4). The pipeline consists of 4 steps: 1. Pre-processing reads, 2. De novo sequence assembly, 3. Linearization and annotation of the draft genome, and 4. Assembly assessment and variant identification. In the first step, reads are processed via several mechanisms. During the pre-processing step, adapters of the form 5’ -AATGATACGGCGACCACCGAGATCTACACTCTTTCCCTACACGACGCTCTTCCGATCT – 3’ are clipped from the reads. Afterward, using a sliding window of 15, reads are dropped if the average phred score in any window dips below 30. Reads are also removed if they map to contaminate reads. In our case, the *Mus musculus* genome (*Mus musculus* GRCm38.p6) was used as the reference to determine contaminate reads. De novo assembly is performed using SSAKE on the clean reads. The default eight trimming and node-overlap parameters were used to construct long contigs which were used as reads for a subsequent assembly using Celera. For linearization and annotation, MHV68 reference genomes (NC_001826.2) were used. The contigs created by Celera were mapped back to the appropriate reference genome where synteny blocks are arranged to linearize the genome. Afterwards, local reassembly and gap closure are performed on gaps to create a final assembled genome. Annotations are transferred to the new assembly and are stored in the appropriate GFF annotation file. Assembly assessment is conducted in a two-step process. First, the pre-processed reads are mapped back to the draft genome to detect polymorphisms in the assembly. Next, pileup information is used to detect coverage for each site in the draft genome. To detect SNPs, we used the Mauve aligner (5) and performed a pairwise alignment of the assembled genomes with their appropriate reference. Since the variant data, as reported in the pipeline, does not detect SNPs from reference, we used this subsequent alignment to obtain SNP information.

References

1. Leang RS, Wu T-T, Hwang S, Liang LT, Tong L, Truong JT, Sun R. 2011. The anti-interferon activity of conserved viral dUTPase ORF54 is essential for an effective MHV-68 infection. PLoS Pathog 7:e1002292.

2. Santana AL, Oldenburg DG, Kirillov V, Malik L, Dong Q, Sinayev R, Marcu KB, White DW, Krug LT. 2017. RTA Occupancy of the Origin of Lytic Replication during Murine Gammaherpesvirus 68 Reactivation from B Cell Latency. Pathogens 6.

3. Quick J, Grubaugh ND, Pullan ST, Claro IM, Smith AD, Gangavarapu K, Oliveira G, Robles-Sikisaka R, Rogers TF, Beutler NA, Burton DR, Lewis-Ximenez LL, de Jesus JG, Giovanetti M, Hill SC, Black A, Bedford T, Carroll MW, Nunes M, Alcantara LC, Sabino EC, Baylis SA, Faria NR, Loose M, Simpson JT, Pybus OG, Andersen KG, Loman NJ. 2017. Multiplex PCR method for MinION and Illumina sequencing of Zika and other virus genomes directly from clinical samples. Nature Protocols 12:1261–1276.

4. Parsons LR, Tafuri YR, Shreve JT, Bowen CD, Shipley MM, Enquist LW, Szpara ML. 2015. Rapid Genome Assembly and Comparison Decode Intrastrain Variation in Human Alphaherpesviruses. mBio 6:e02213-14.

5. Darling ACE, Mau B, Blattner FR, Perna NT. 2004. Mauve: multiple alignment of conserved genomic sequence with rearrangements. Genome Res 14:1394–1403.
